# Mitochondrial genome of *Isatis indigotica* reveals repeat-mediated recombination and phylogenetic insights in *Cruciferae*

**DOI:** 10.3389/fpls.2025.1655810

**Published:** 2025-10-15

**Authors:** Shaoshuai Yu, Meiling Qin, Emmanuel Fleming, Xun Gong, Min Tang

**Affiliations:** ^1^ Department of Pharmacy, Affiliated People’s Hospital of Jiangsu University, Zhenjiang, Jiangsu, China; ^2^ School of Life Sciences, Jiangsu University, Zhenjiang, Jiangsu, China; ^3^ Department of Rheumatology and Immunology, Affiliated Hospital of Jiangsu University, Zhenjiang, Jiangsu, China

**Keywords:** *Isatis indigotica*, mitogenome, repeat-mediated recombination, RNA-editing site, Brassicaceae

## Abstract

*Isatis indigotica* is an important medicinal plant extensively used in traditional Chinese medicine for its antiviral and anti-inflammatory properties. While previous studies have elucidated its nuclear and plastid genomes, the mitochondrial genome (mitogenome) —critical for understanding organellar evolution, intracellular DNA transfer, and stress response mechanisms—has remained uncharacterized. Here, we present a complete *de novo* assembly and comprehensive analysis of the *I. indigotica* mitogenome, generated using high-fidelity long-read sequencing technologies. The circular mitogenome spans 260,864 bp and encodes 31 protein-coding genes, 21 transfer RNAs, and 3 ribosomal RNAs. Repetitive sequences constitute 12.3% of the genome, with large repeats mediating homologous recombination and generating alternative conformations. A total of 488 RNA editing sites were identified, predominantly of the cytidine-to-uridine (C-to-U) type, indicating extensive post-transcriptional modification. We also detected 36 regions homologous to the plastid genome, reflecting active inter-organellar DNA transfer. Codon usage analysis revealed a preference for A/U-ending codons, and Ka/Ks analysis suggested strong purifying selection in most mitochondrial genes. Phylogenomic analysis based on 24 conserved mitochondrial genes placed *I. indigotica* in close proximity to *Brassica* species, supporting its taxonomic placement within the Brassicaceae family and aligning with plastid-based phylogenies. This study provides the first complete mitogenome of *I. indigotica*, offering valuable insights into mitogenome architecture, RNA editing dynamics, and plastid–mitochondrial interactions, while contributing to broader evolutionary and genomic understanding of cruciferous medicinal plants.

## Introduction

*Isatis indigotica* Fortune, a biennial herb of the *Brassicaceae* (*Cruciferae*) family, has been widely cultivated in East Asia, particularly China, for both its medicinal and economic value (Chen et al., 2014; Shen et al., 2024). Known in traditional Chinese medicine as the source of *Isatidis Radix* and *Isatidis Folium*, its roots and leaves are used to treat febrile diseases, viral infections, and inflammatory conditions (Kong et al., 2008; Ding and Zhu, 2020; Sun et al., 2021). Phytochemical studies have identified a wealth of bioactive compounds including alkaloids, lignans, and flavonoids, contributing to its pharmacological efficacy. Recent functional analysis has further elucidated key glycosyltransferase genes involved in the biosynthesis of flavonoid glycosides, emphasizing the role of specialized metabolites in the plant’s therapeutic profile (Tan et al., 2025). Despite its medicinal prominence, the taxonomic identity of *I. indigotica* has long been confounded with that of *Isatis tinctoria*, a European congener with historical relevance in dye production (Lu et al., 2018; Speranza et al., 2020). Chloroplast genome (cpgenome or cpDNA) comparison has provided molecular evidence distinguishing these species, with specific divergence in gene content and structure supporting their independent evolutionary lineages (Zhu et al., 2021; Wang et al., 2022; Zhang et al., 2022). Moreover, a chromosome-scale assembly has revealed a complex genomic architecture and biosynthetic gene clusters underpinning its medicinal traits, offering new resources for functional genomics (Kang et al., 2020).

Although nuclear and plastid genomes of *I. indigotica* have been increasingly characterized, its mitochondrial genome (mitogenome or mtDNA) remains unexplored. This presents a critical gap, as plant mitogenomes are not only crucial for cellular energy metabolism but are also recognized for their unique genomic properties—large size, high structural plasticity, and the prevalence of repeat-mediated homologous recombination (Liu et al., 2025; Wang et al., 2025; Zhou et al., 2025). Such recombination events can lead to genome rearrangements, multi-chromosomal configurations, and gene expression variability. In *Brassicaceae*, mitochondrial variation has been implicated in cytoplasmic male sterility, adaptive evolution, and interspecies hybridization (Chen et al., 2025; Jiang et al., 2025; Voisin et al., 2025; Zhao et al., 2025). The lack of mitochondrial data for *I. indigotica* limits phylogenetic resolution and hinders organelle-level evolutionary inferences, especially when nuclear and plastid markers show conflicting signals. Notably, chloroplast phylogenetic analysis has revealed a close sister relationship between *I. indigotica* and *Raphanus sativus* within the *Brassicaceae*, as evidenced by cpgenome data. This placement, while taxonomically consistent, warrants further validation using complementary data such as mitogenomes (Yang and Wang, 2017).

Plant mitochondrial genomes are complex and exhibit significant variability in both size and structure (Chevigny et al., 2020; Arimura and Nakazato, 2024; Forner, 2025). These genomes are essential for energy production, housing genes responsible for key components of respiration. While the gene content is relatively minimal, the majority of the genome consists of non-coding regions that contribute to its dynamic structure. This flexibility allows for frequent rearrangements and the creation of multiple subgenomes within a single mitochondrion. Despite this structural instability, the gene sequences in plant mitochondrial genomes evolve slowly due to efficient repair mechanisms, particularly homologous recombination, which helps maintain genome integrity (Zwonitzer et al., 2024). The variability in the number of mitochondria and the genome copies within a cell further influences mitochondrial function. Recent advancements in genetic engineering tools have enabled precise modifications to mitochondrial DNA, providing new opportunities to study mitochondrial roles in plants and improve agricultural traits. In this study, we assembled and annotated the complete mitogenome of *I. indigotica* using high-fidelity (HiFi) long-read sequencing, and systematically analyzed its structural features, repeat elements, RNA editing sites, and phylogenetic position. Particular attention was paid to repeat-mediated recombination events and organellar DNA transfer, with the goal of elucidating the dynamic architecture and evolutionary trajectory of the mitogenome. The resulting data provide not only molecular tools for resolving taxonomic boundaries within *Isatis*, but also broader insights into mitogenome evolution across *Brassicaceae*.

## Materials and methods

### High-integrity DNA extraction and long-read sequencing

**Figure 1:**
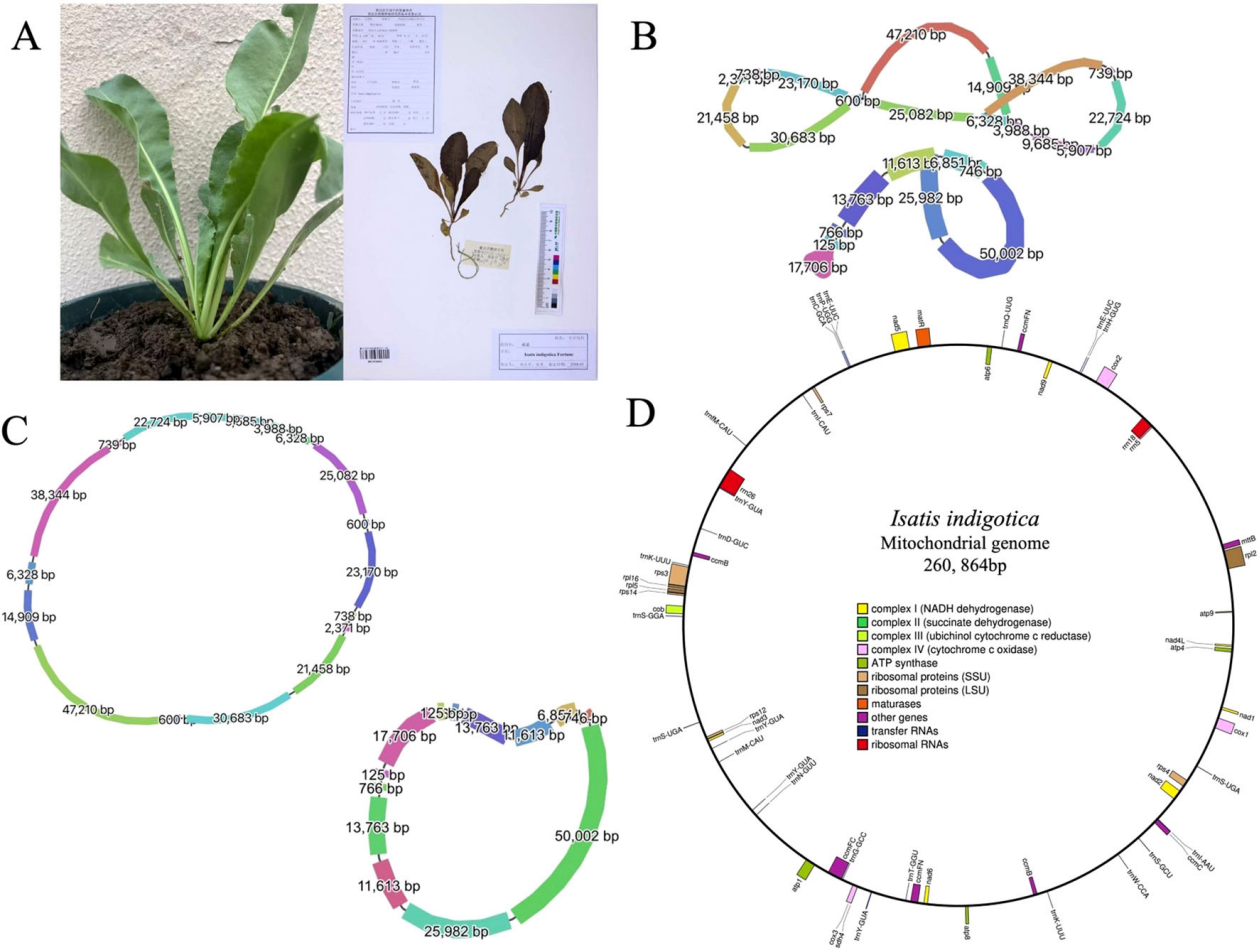
Morphological characteristics and organelle genome structures of *I. indigotica*. **(A)** Photograph of a living *I. indigotica* plant (left) and its herbarium specimen (right). **(B)** Graphical representation of the multipartite structure of the mitogenome (top) and cpgenome (bottom) of *I. indigotica*, with segment lengths (bp) labeled. Line thickness reflects relative sequencing depth, with thicker lines indicating higher coverage. **(C)** Circular maps of the mitogenome (top) and cpgenome (bottom), showing the lengths of each genomic segment. **(D)** Gene map of the complete mitogenome of *I. indigotica*, illustrating gene categories including NADH dehydrogenase, succinate dehydrogenase, cytochrome oxidase, ATP synthase, ribosomal proteins, maturases, tRNAs, and rRNAs.

Young tender leaves of *I. indigotica* were sourced directly from Xianglian Horticulture, a verified commercial nursery located in Weitang Town, Xiangcheng District, Suzhou, China (31.2250° N, 120.6420° E) (**Figure 1A**). The identity of the entire plant was initially verified in-house by comparing morphological characteristics to the reference specimen image IMC0058462 from the National Plant Specimen Resource Center of China Digital Herbarium (NPSRCCDH). To reinforce the accuracy of this species determination, two independent experts from Jiangsu University—both with extensive experience in plant taxonomy—were subsequently consulted and confirmed the identification. Young leaves were freshly collected directly from potted *I. indigotica* plants and immediately rinsed with DEPC-treated water. The cleaned tissue was flash-frozen in liquid nitrogen and stored at −80 °C to preserve DNA integrity. High-molecular-weight genomic DNA was extracted using a modified CTAB protocol optimized for plant materials. DNA quality was assessed by 0.75% agarose gel electrophoresis, while purity and concentration were determined using both UV spectrophotometry (NanoDrop One, Thermo Fisher Scientific) and fluorometry (Qubit 3.0, Life Technologies). Only high-quality DNA was used for library construction with the SMRTbell Express Template Preparation Kit 2.0 (Pacific Biosciences), followed by long-read sequencing on the PacBio Sequel II system for accurate organelle genome assembly.

### Graph-based assembly and standardized annotation of mitogenome from high-fidelity reads

The mitogenome of *I. indigotica* was assembled using a multi-stage pipeline optimized for PacBio HiFi reads. To identify mitochondrial-derived sequences, HiFi reads were first aligned against three published *Brassicaceae* mitochondrial reference genomes: *Brassica rapa* (NC_049892.1) (Ren et al., 2023), *R. sativus* (NC_018551.1) (Chang et al., 2013), and *Sinapis arvensis* (KM851044.1) (Sang et al., 2020). Prior to alignment, BLAST databases were constructed for each reference using makeblastdb from BLAST+ (v2.13.0+), with default parameters and nucleotide indexing (Camacho et al., 2009). Read alignment was then conducted using blastn with the following parameters: -evalue 1e-5 -outfmt 6 -max_hsps 10 -word_size 7 -task blastn-short. The resulting alignments were filtered to retain only uniquely matched reads, and read IDs from all three comparisons were merged to generate a non-redundant mitochondrial candidate set. These filtered reads were extracted using seqkit grep and subsequently assembled *de novo* with PMAT (Plant Mitochondrial Assembler Tool), employing the autoMito module with -st hifi mode, an estimated genome size of 300 Mb, and 24 computational threads. To improve the accuracy of the long-read data, PMAT, through its “correct_sequences.py” script, calls the Canu program to perform error correction, utilizing overlap-based assembly algorithms and error correction techniques. This procedure yielded a complete, circularized mitogenome of *I. indigotica* suitable for downstream analysis. To evaluate the assembly structure, the graphical layout in GFA format was inspected using Bandage (v0.8.1), allowing visual confirmation of the circular topology and connection paths among contigs (Wick et al., 2015).

After genome assembly, structural gene annotation was carried out using the Plant Mitochondrial Genome Annotator (PMGA, v1.5.3), a specialized tool designed for organelle genome annotation workflows (Li et al., 2025). The software was deployed within a reproducible computational environment managed by mamba, and executed inside a containerized system based on singularity (v3.7.2), ensuring compatibility and scalability across platforms. To improve annotation fidelity, the internal reference dataset (Dataset1) bundled with PMGA was utilized, offering curated models of mitochondrial genes. The initial automated annotation output was carefully reviewed and refined using Apollo (v2.5.0) (Lee et al., 2013), with manual correction of ambiguous gene boundaries and confirmation of structural features. The resulting high-confidence GFF3 annotation file, after manual curation, was submitted along with the corresponding FASTA genome sequence to the GenBase database (https://ngdc.cncb.ac.cn/genbase/?lang=en) for public access and future reference (Bu et al., 2024).

### Computational detection and experimental assessment of mitochondrial repeats and recombination

Repetitive sequences in eukaryotic genomes are broadly classified into simple sequence repeats (SSRs), tandem repeats, and dispersed repeats based on their structural features and genomic organization (Han et al., 2022; Ala et al., 2023; Li et al., 2023; Zhang et al., 2023; Wang et al., 2024). In this study, SSRs in the mitogenome of *I. indigotica* were identified using the misa.pl script (v2.1) with predefined thresholds: a minimum of 10 repeat units for mononucleotides, 5 for dinucleotides, and 4, 3, 3, and 3 for tri-, tetra-, penta-, and hexanucleotides, respectively (Thiel et al., 2003). A maximum distance of 1,000 bp was allowed between neighboring SSRs to detect potential compound repeats. Tandem repeats were identified using Tandem Repeats Finder (TRF, v4.09) under the parameter setting “2 7 7 80 10 50 500 -f -d -m”, which is optimized for the detection of tandem motifs in organelle genomes (Benson, 2023; Zhang et al., 2024). Dispersed repeats, defined as non-contiguous homologous sequences, were detected using the REPuter tool with the following parameters: minimum repeat length of 30 bp, maximum of 500 repeats per genome, and a Hamming distance threshold of 3 to accommodate minor mismatches (Kurtz et al., 2023). To gain insights into the distribution and organization of repeat elements across the mitogenome, all detected SSRs, tandem repeats, and dispersed repeats were mapped using Circos software (v0.69-8) (Krzywinski et al., 2023).

To assess whether dispersed repeat pairs mediate homologous recombination, two structural models were constructed for each candidate pair: (i) a reference configuration representing the native genomic arrangement, and (ii) a recombinant configuration simulating the outcome of recombination (Gualberto et al., 2014; Gualberto and Newton, 2017). For each model, 1000 bp flanking sequences surrounding the repeat pair were extracted. Nanopore long reads were then mapped to both models, and support for the recombinant structure was considered indicative of active recombination at that locus.

**Figure 2:**
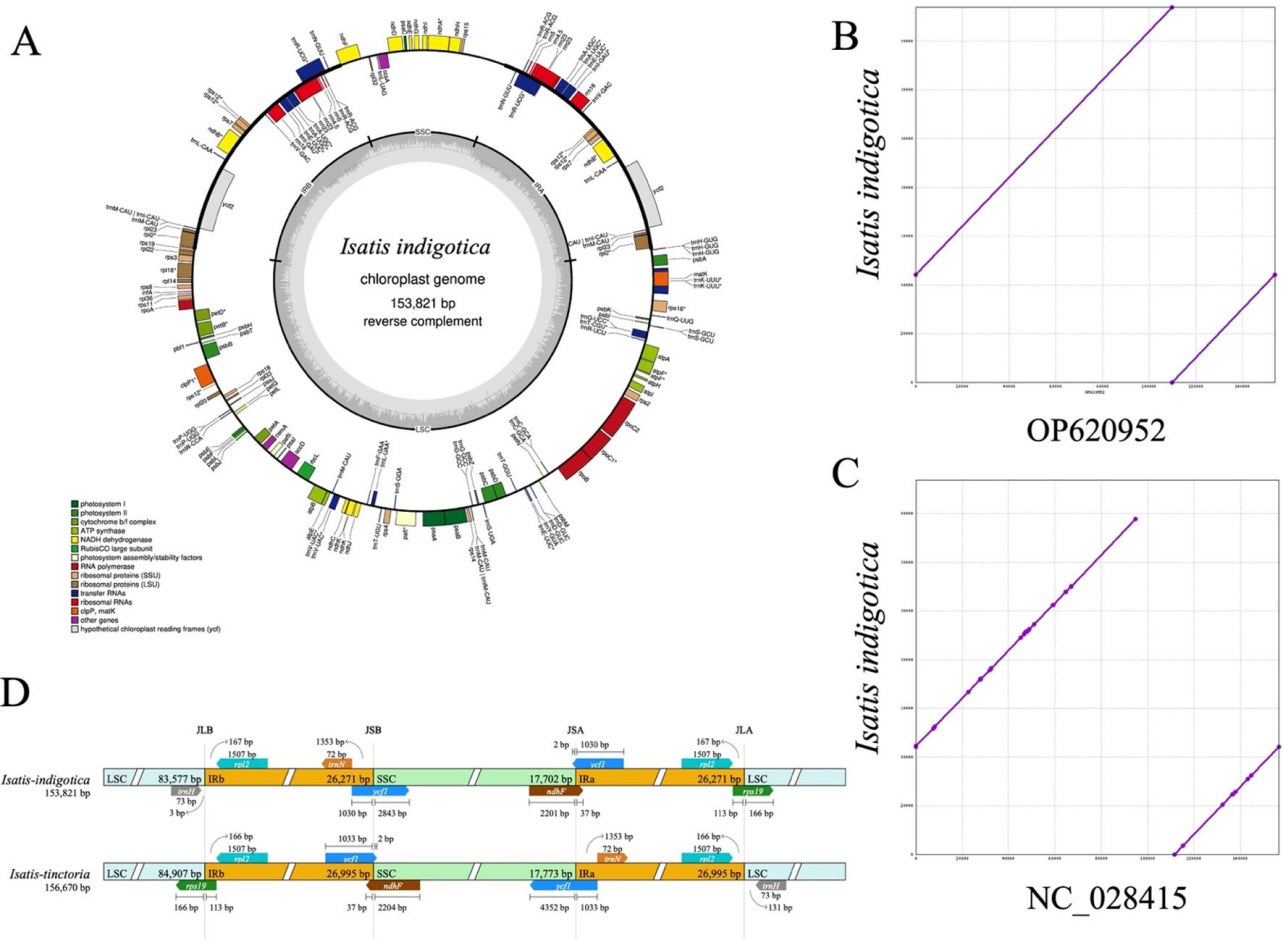
Structural and comparative analysis of the cpgenome of *I. indigotica.* **(A)** Circular map of the *I. indigotica* cpgenome (153,821 bp), with genes color-coded by functional category, illustrating the structural organization and annotation. **(B)** Sequence alignment of the *I. indigotica* cpgenome with accession OP620952 (same species) reveals near-complete sequence identity. **(C)** Comparative alignment with accession NC_028415 (*I. tinctoria*) shows minor sequence variations, reflecting interspecific genomic differences. **(D)** Structural comparison of the LSC, SSC, and IR boundary regions between *I. indigotica* and *I. tinctoria*, highlighting differences in boundary positioning and segment lengths.

To experimentally validate selected recombination sites, PCR amplification was performed using primers designed to span the flanking regions of the repeat pair. Specifically, primers were designed in the 1000 bp regions upstream and downstream of each repeat: a forward primer (F) in the upstream region and a reverse primer (R) in the downstream region. For dispersed repeat pairs that are located far apart in the mitogenome, two sets of primers were used: F1/R1 and F2/R2 for the two respective repeat loci. If direct (forward) recombination occurs, it can be detected by amplification of bands using the F1/R2 and F2/R1 primer combinations. Conversely, if inverted recombination occurs, it may be evidenced by amplification with F1/F1 or R1/R2 combinations. These diagnostic PCR patterns provide direct molecular evidence for the type and presence of recombination events mediated by repeat sequences [refer to **Figure 2** in (Liu et al., 2025)].

Each 25 μl PCR reaction contained 1 μl genomic DNA, 1 μl each of 8 μM forward and reverse primers, 13 μl of 2× Taq PCR Master Mix, and 10 μl nuclease-free water. Thermocycling conditions were as follows: 96 °C for 3 min initial denaturation; 32 cycles of 94 °C for 30 s, 62 °C for 30 s, and 72 °C for 1 min; and a final extension at 72 °C for 10 min. PCR products were analyzed by agarose gel electrophoresis, and positive bands were sequenced using the Sanger method to confirm recombination events and evaluate repeat-associated structural variation.

### Cpgenome recovery and annotation

During the structural correction phase of mitogenome assembly, the complete cpgenome of *I. indigotica* was incidentally resolved as an independent circular contig within the graphical assembly output. This structure was identified and separated using Bandage (v0.8.1) through examination of the GFA-format assembly graph generated from long-read sequencing data (**Figures 1B,C**). The circular chloroplast sequence was subsequently exported in FASTA format for further characterization.

Annotation of the cpgenome was conducted using the GeSeq web server, with *I. indigotica* Fortune employed as the reference genome to guide gene prediction and structural annotation (GenBank: KT939360) (Yang and Wang, 2017). The annotated cpgenome was visualized using OGDRAW (v1.3.1) (Greiner et al., 2019), resulting in a high-resolution circular genome map. The generated GFF3 file was subsequently curated manually to correct annotation discrepancies and feature boundaries. Both the finalized GFF3 and the corresponding FASTA file were submitted to the GenBase database for long-term archival and community access.

Species-level validation of the cpgenome was performed through comparative analysis with two previously published plastomes: *I. indigotica* (GenBank: OP620952) and *I. tinctoria* (GenBank: NC_028415), confirming its taxonomic identity.

### Detection of mitochondrial-plastid and nuclear DNA transfer events

To investigate potential sequence transfer events between genomic compartments in *I. indigotica*, a comprehensive analysis of mitochondrial-plastid DNA transfer (MTPT) was conducted. The analysis employed BLASTn (v2.13.0) with the plastid genome used as the query and the mitogenome as the target. A stringent e-value cutoff of 1e-6 was set to ensure the detection of only high-confidence homologous regions (Chen et al., 2015). Regions of plastid origin identified within the mitogenome were visualized using TBtools (v2.010). The corresponding sequences were further annotated via GeSeq to assign functional roles and validate their organellar origin (Chen et al., 2020).

In parallel, the nuclear genome of *I. indigotica* (GenBank accession: VHIU00000000), which comprises seven chromosomes in its haploid complement, was also analyzed to assess possible organelle-to-nucleus DNA integration. This tri-compartmental comparative approach enabled the identification and characterization of both plastid-to-mitochondria and organelle-to-nucleus horizontal sequence transfers, offering insights into the dynamic genome interactions and evolutionary history of the species.

### Dual-strategy identification of mitochondrial RNA editing sites and codon usage bias analysis

RNA editing is a crucial post-transcriptional process in plant mitochondria, predominantly characterized by cytidine-to-uridine (C-to-U) conversions. These events can restore conserved amino acids, generate functional start or stop codons, and impact mitochondrial protein function and stability (Lukes et al., 2021). To comprehensively identify RNA editing events in *I. indigotica*, we employed two complementary strategies: (1) RNA-seq read mapping and variant calling, and (2) comparative prediction using conserved reference genomes.

For the mapping-based approach, publicly available RNA-seq data (SRA: SRR9329298, BioProject: PRJNA549758) were aligned to a custom-assembled mitogenome of *I. indigotica* (genbase: C_AA108663) (Kang et al., 2020). The processing pipeline consisted of fastp (v0.24.0) for adapter removal and quality filtering, fastQC (v0.12.1) for quality assessment, HISAT2 (v2.2.1) for splice-aware alignment, and SAMtools (v1.6) for BAM file handling (Li et al., 2009; Kim et al., 2015; Chen et al., 2018; Wingett and Andrews, 2018). Variant calling was performed using BCFtools (v1.21), and RNA editing candidates were filtered using custom scripts developed in-house (Narasimhan et al., 2016; Danecek and McCarthy, 2017; Genovese et al., 2024). In parallel, a reference-based prediction strategy was implemented using the PREPACT3 platform (http://www.prepact.de/prepact-main.php, accessed on 26 Jun 2025; version 3.12.0/2.2.26+) (Lenz et al., 2010). To maintain high specificity, only two mitochondrial reference genomes were used: *Brassica napus* (NC_008285.1) and *Citrullus lanatus* (NC_014043.1). A stringent BLASTX e-value threshold of 1e−3 was applied to identify conserved editing sites with high confidence, following established methodologies (Lenz and Knoop, 2013).

Codon usage optimization—defined as the preferential employment of synonymous codons to improve translational fidelity and effectiveness—is a recognized phenomenon in both unicellular and multicellular organisms (Presnyak et al., 2015; Sen et al., 2020). This codon usage bias arises from a combination of selective pressures, species-specific translational demands, and random genetic events (Hanson and Coller, 2018). In this study, mitochondrial protein-coding proteins (PCGs) of *Eleutherococcus senticosus* were subjected to codon bias evaluation. The analysis was conducted using TBtools software (v2.010), and relative synonymous codon usage (RSCU) values were determined via CodonW (v1.4.4) (Charif et al., 2005).

### Evolutionary rate and nucleotide diversity analyses of mitochondrial PCGs in *I. indigotica* and related *Brassicales* species

Assessing the evolutionary patterns of PCGs is essential for understanding molecular adaptation and divergence among closely related species. In this study, the rates of nonsynonymous (Ka) and synonymous (Ks) substitutions were estimated to evaluate the selective pressures acting on mitochondrial PCGs of *I. indigotica* in comparison with 15 other representative species from the order Brassicales. The Ka/Ks ratio serves as an indicator of selection: values >1 suggest positive selection, = 1 imply neutral evolution, and <1 indicate purifying selection.

Homologous gene pairs between *I. indigotica* and the other Brassicales species were identified using BLASTN (v2.10.1). Shared mitochondrial PCGs were aligned using MAFFT (v7.313) in auto mode to ensure accurate multiple sequence alignment (Rozewicki et al., 2019). Ka and Ks values were computed with the Maximum Likelihood (MLWL) method implemented in Ka/Ks Calculator v2.0 (Zhang et al., 2006; Wang et al., 2010; Zhang, 2022). The distribution of Ka/Ks ratios across genes was visualized using boxplots generated with the R package *ggplot2*. In parallel, to assess genetic variability at the nucleotide level, aligned homologous gene sequences were analyzed using DnaSP v5, and nucleotide diversity (Pi) values were calculated for each PCG (Rozas et al., 2003; Librado and Rozas, 2009; Rozas et al., 2017). This analysis provides insights into the extent of sequence polymorphism within mitochondrial genes, further contributing to our understanding of evolutionary constraints and divergence patterns across Brassicales mitogenomes.

### Phylogenomic reconstruction and synteny mapping of *I. indigotica* mitogenome

To elucidate the phylogenetic position of *I. indigotica* within angiosperms, a comparative mitogenomic analysis was performed using mitogenome sequences from 26 plant species representing four taxonomic orders. One species from the order Solanales was included as the outgroup to root the phylogenetic tree (**Supplementary Table S1**). Among the selected species, 16 belonged to the family *Brassicaceae*, including *I. indigotica* itself. These cruciferous taxa were: *Arabidopsis thaliana*, *Arabidopsis lyrata*, *Boechera stricta*, *Brassica napus*, *B. rapa*, *Brassica oleracea*, *Brassica juncea*, *Brassica carinata*, *Capsella rubella*, *Crucihimalaya lasiocarpa*, *Descurainia sophia*, *I. tinctoria*, *Lepidium apetalum*, *Lepidium sativum*, and *R. sativus*. To provide broader phylogenetic context, 10 additional non-*Brassicaceae* species were included: *Apium graveolens* (Apiaceae), *Panax quinquefolius* (Araliaceae), *Phaseolus vulgaris*, *Aeschynomene indica*, *Senna tora*, and *Vigna angularis* (all Fabaceae), *Saussurea costus* and *Taraxacum mongolicum* (Asteraceae), and the outgroup *Solanum aethiopicum* (Solanaceae). This diverse taxon set enabled robust resolution of both intra- and inter-family evolutionary relationships.

All genomic datasets were retrieved from GenBank and subsequently processed using PhyloSuite (v1.2.3), which facilitated the extraction and reformatting of mitochondrial coding sequences (Zhang et al., 2020; Xiang et al., 2023). Multiple sequence alignment (MSA) of conserved gene regions was performed using MAFFT (v7.313) to ensure high-accuracy alignment across all taxa involved (Rozewicki et al., 2019). To determine the most appropriate nucleotide substitution models for downstream phylogenetic inference, PartitionFinder2 was employed. Maximum likelihood phylogenetic reconstruction was carried out using IQ-TREE2 (v2.1.4) with ultrafast bootstrap support (Minh et al., 2020). The selection of the best nucleotide substitution models was based on the Akaike Information Criterion (AIC) and Bayesian Information Criterion (BIC), which evaluated various models to identify the one that best fit the data. The final result indicated that the GTR+F+I+G4 model was the most appropriate, as it provided the lowest log-likelihood value and the best fit according to both AIC and BIC criteria. The final tree visualization and annotation were achieved through iTOL (v6), offering customizable graphics for enhanced biological interpretation (Letunic and Bork, 2021).

To investigate mitogenome collinearity and structural conservation in *I. indigotica*, three additional mitogenomes—*Apium graveolens*, *Taraxacum mongolicum*, and *Vigna angularis*—were selected for comparative analysis. These species, representing different families within the eudicots, provided a broad phylogenetic context for assessing syntenic relationships. Pairwise comparisons were conducted using BLASTn, with an e-value cutoff of 1e-6 to ensure specificity. Homologous regions exceeding 500 bp in length were designated as conserved collinear blocks. The collinearity relationships among *I. indigotica*, *I. tinctoria*, and the three selected eudicot species were visualized using NGenomeSyn (v1.41), enabling detailed inspection of genome rearrangements and conserved structural segments across lineages (He et al., 2023).

To assess large-scale sequence conservation and structural variation among closely related cruciferous species, comparative alignment of mitogenomes from *I. indigotica*, *I. tinctoria*, and *R. sativus* was conducted. Whole-genome alignments were generated using long-read-assembled mitogenomes and visualized with Mauve (v2.4.0) alignment software (https://sourceforge.net/projects/mauve). The Mauve viewer enabled the detection of locally collinear blocks (LCBs) and facilitated inspection of genome rearrangements, inversions, and insertions/deletions across the three *Brassicaceae* species.

## Results

### Data generation and genome assembly

A total of 368,520 HiFi long reads were generated using the PacBio Revio platform, producing approximately 6.48 Gb of high-quality sequencing data. The mean read length was 17,596 bp, with a median length of 16,546 bp and a read length N50 of 17,479 bp, indicating a highly consistent and uniform long-read distribution across the dataset (**Supplementary Figure S1A**). The median read quality reached Q33, demonstrating the exceptional base-level accuracy achieved through PacBio’s circular consensus sequencing (CCS) technology (**Supplementary Figure S1B**). The sequencing depth for the mitochondrial genome was approximately 24.9x, and for the chloroplast genome, the sequencing depth was approximately 42.2x, ensuring comprehensive coverage of both genomes. To ensure data transparency and facilitate future reuse, the raw HiFi sequencing data have been deposited in the Genome Sequence Archive (GSA) at the National Genomics Data Center (NGDC) under accession number CRA026873 (https://ngdc.cncb.ac.cn/gsa) (Members and Partners, 2025). Furthermore, the annotated mitogenome assembly has been submitted to GenBase, also hosted by NGDC/CNCB, and is accessible under accession number C_AA108663.1 (https://ngdc.cncb.ac.cn/genbase) (Bu et al., 2024). The final assembly comprises a complete circular mitogenome of 260, 864 base pairs in length (**Figure 1D**).

### Mitogenome classification and evolutionary features of mitochondrial genes

The mitogenome of *I. indigotica*, as annotated using the PMGA pipeline, comprises 65 unique genes, including 23 core PCGs, 9 variable genes, 3 ribosomal RNA (rRNA) genes, and 18 distinct transfer RNA (tRNA) species, with copy number variation observed in several tRNAs (**Table 1**). The core PCGs are involved in essential mitochondrial functions and include five ATP synthase subunits (*atp1, atp4, atp6, atp8, atp9*), nine NADH dehydrogenase genes (*nad1, nad2, nad3, nad4, nad4L, nad5, nad6, nad7, nad9*), one ubiquinol cytochrome c reductase gene (*cob*), and six genes related to cytochrome c biogenesis, with duplicated copies of *ccmB* and *ccmFN* (*ccmB* ×2*, ccmC, ccmFC, ccmFN* ×2). Three cytochrome c oxidase genes (*cox1, cox2, cox3*) and one maturase gene (*matR*) were also identified.

**Table 1:** Categorization of mitochondrial genes identified in *I. indigotica*.

Gene classification	Functional role	Gene list
Core genes	ATP synthase	*atp1*, *atp4*, *atp6, atp8, atp9*
	NADH dehydrogenase	*nad1*, *nad2*, *nad3*, *nad4*, *nad4L*, *nad5*, *nad6*, *nad7*, *nad9*
	Ubichinol cytochrome creductase	*Cob*
	Cytochrome *c* biogenesis	*ccmB* (x2), *ccmC*, *ccmFC*, *ccmFN* (x2)
	Cytochrome *c* oxidase	*cox1*, *cox2*, *cox3*
	Maturases	*matR*
Variable genes	Large subunit of ribosome (LSU)	*rbl2*, *rbl5*, rpl16^*^
	Small subunit of ribosome (SSU)	*rps3*, *rps4*, *rps7*, rps12^*^, rps14^*^
	Succinate dehydrogenase	*sdh4*
	Membrane transport	*mttB* (*tatB*)^#^
rRNA genes	Ribosome RNA	*rrn5*, *rrn18*, *rrn26*
tRNA genes	Transfer RNA	*trnC-GCA*, *trnD-GUC*, *trnE-UUC*(x2), *trnG-GCC*, *trnI-AAU, trnK-UUU*(x2), *trnM-CAU*(x3), *trnN-GUU*, *trnP-UGG*, *trnQ-UUG*, *trnS-GCU*, *trnS-GGA*, *trnS-UGA*(x2), *trnT-GGU*, *trnW-CCA*, *trnY-GUA*(x4), *trnH-GUG*

*The numbers in parentheses represent gene copy numbers when applicable. Asterisks* (*) denote genes with variable presence across angiosperm mitogenomes, or those showing signs of pseudogenization or dual genomic localization (Sun et al., 2018; Liu et al., 2020; Gao et al., 2022; Xiang et al., 2022). The hash symbol (#) marks genes that are rarely observed in land plant mitochondria; their annotation may be tentative and potentially of prokaryotic or horizontally transferred origin.

he mitochondrial genes of *I. indigotica* are classified into core functional groups (ATP synthase, NADH dehydrogenase, ubiquinol cytochrome c reductase, cytochrome c biogenesis, cytochrome c oxidase, and maturases), variable genes (including ribosomal protein genes of the large and small subunits, succinate dehydrogenase, and membrane transport proteins), plastid-derived sequences, ribosomal RNA genes, and transfer RNA genes.

The variable gene set consists of three large ribosomal subunit genes (*rpl2, rpl5, rpl16*) and five small ribosomal subunit genes (*rps3, rps4, rps7, rps12, rps14*). Notably, *rps12*, *rps14*, and *rpl16* are variably retained in angiosperm mitogenomes and are often considered non-canonical or plastid-derived, suggesting possible intracellular gene transfer or pseudogenization. The genome also encodes *sdh4*, a succinate dehydrogenase subunit, and *mttB*, a putative membrane transporter gene homologous to bacterial *tatB*, which may represent a case of horizontal gene transfer. The rRNA gene complement includes *rrn5*, *rrn18*, and *rrn26*, which constitute the core structural components of mitochondrial ribosomes. The tRNA set contains 18 distinct species, with multiple copies observed for *trnE-UUC* (×2), *trnK-UUU* (×2), *trnM-CAU* (×3), *trnS-UGA* (×2), and *trnY-GUA* (×4), providing redundancy typical of plant mitochondrial translation systems.

### Synonymous codon preference in mitochondrial PCGs

**Figure 3:**
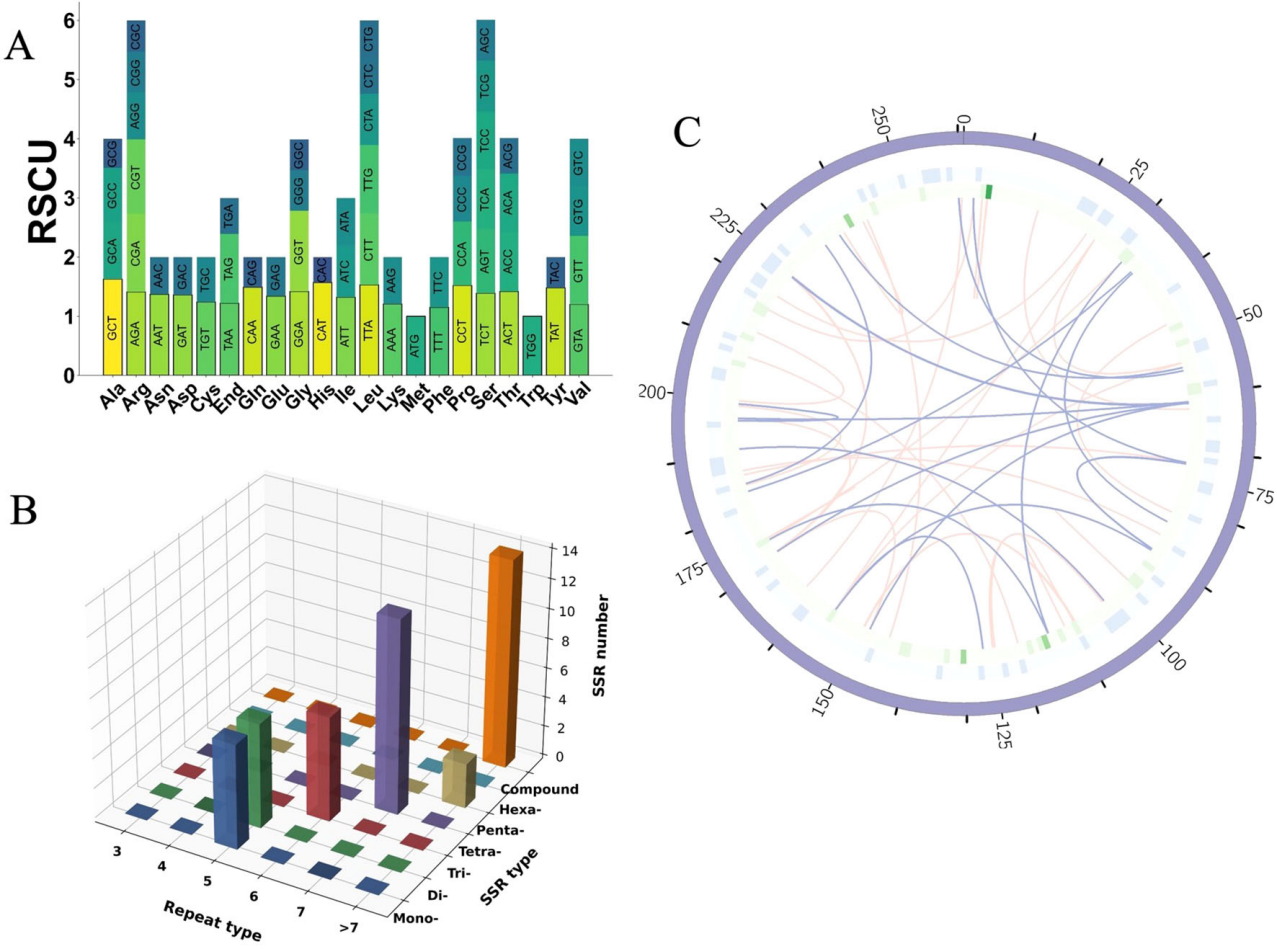
Codon usage, SSR analysis, and repeat element distribution in the mitogenome of *I. indigotica*. **(A)** RSCU values for PCGs in the mitogenome of *I. indigotica*. **(B)** Distribution of SSRs in the mitogenome of *I. indigotica*, categorized by repeat type and SSR type. **(C)** Distribution of repeat elements in the mitogenome of *I. indigotica*. The outermost circle represents SSRs, the middle circle shows tandem repeats, and the innermost circle illustrates dispersed repeats, with pink lines indicating forward repeats and purple lines indicating palindromic repeats.

The mitochondrial PCGs of *I indigotica* display discernible codon usage biases, providing insights into the evolutionary dynamics and translational regulation within the mitogenome. A total of 61 sense codons encode the standard 20 amino acids, with the exception of methionine and tryptophan, both of which are encoded by single codons (**Figure 3A**). The distribution of relative synonymous codon usage (RSCU) values indicates varying degrees of codon preference, likely shaped by a combination of mutational biases, selective constraints, and tRNA availability.

Codons with RSCU values greater than 1 are considered preferentially used. Examples include GCT (alanine, 1.63), CGT (arginine, 1.26), AGA (arginine, 1.41), AAT (asparagine, 1.37), GAT (aspartic acid, 1.36), and TGT (cysteine, 1.24), suggesting favored usage during mitochondrial translation. In contrast, codons such as GCG (alanine, 0.49), CGG (arginine, 0.69), CGC (arginine, 0.53), AAC (asparagine, 0.63), and GAC (aspartic acid, 0.64) are underrepresented, with RSCU values below 0.7 (**Supplementary Table S2**). These patterns collectively suggest a moderate codon usage bias in *I. indigotica*, with a tendency toward A- and T-ending codons at the third position, consistent with observations in other angiosperm mitogenomes.

### Comprehensive profiling of repetitive elements in mitogenome

A comprehensive analysis of SSRs in the mitogenome of *I. indigotica* revealed 51 distinct SSR loci, with repeat unit lengths ranging from 10 to 1,451 base pairs (**Figure 3B**). These SSRs were categorized into seven types—mononucleotide (p1), dinucleotide (p2), trinucleotide (p3), tetranucleotide (p4), pentanucleotide (p5), hexanucleotide (p6), and compound repeats (c)—and further grouped by repeat unit numbers (three to >7). Among them, compound SSRs were the most abundant (n = 14), followed by tetranucleotide repeats (n = 13) and dinucleotide repeats (n = 8) (**Supplementary Table S3**). Mononucleotide and trinucleotide motifs were observed six times each, while pentanucleotide and hexanucleotide repeats were less frequent, with only two and one instance(s), respectively. The high prevalence of compound and tetranucleotide SSRs suggests their potential role in promoting genomic plasticity and structural variability. These SSRs were visualized by repeat type and length category in a 3D bar plot.

In addition to SSRs, other repetitive elements were also analyzed, including dispersed and tandem repeats (**Supplementary Table S4**). A total of 396 dispersed repeats were identified, comprising 216 forward repeats and 180 palindromic repeats, with repeat lengths ranging from 30 bp to 11,396 bp. Tandem repeats were also detected, though less abundant. The spatial organization of these three major repeat types—SSRs (outermost circle), tandem repeats (middle circle), and dispersed repeats (innermost circle)—is illustrated in **Figure 3C**. In this circular map, green bars indicate SSR loci, light blue boxes represent tandem repeats, and arcs connect dispersed repeat pairs, with pink lines denoting forward repeats and purple lines indicating palindromic repeats. This layered distribution provides insight into the repeat architecture and potential recombinogenic regions across the *I. indigotica* mitogenome.

### Repeat-mediated structural rearrangements in mitogenome

To experimentally validate homologous recombination events facilitated by repetitive elements, five repeat pairs—two forward repeats (F96 and F146) and three palindromic repeats (P93, P134, and P326)—were selected for PCR-based analysis (**Supplementary Table S5**). Primers were designed to flank each repeat with ~100 bp of adjacent sequence. Two sets of primer combinations (F1/R1 and F2/R2) targeted the native configurations of each repeat region, while cross-combinations (F1/R2 and F2/R1) were designed to detect recombined conformations (**Table 2**).

**Table 2:** Primer sequences and PCR conditions for verification of homologous recombination mediated by repetitive sequences.

Repeats	Primers	Sequence 5’-3’	Product length of F1R1 and F2R2 (bp)	PCR procedure
Forward Repeat F96	F96-F1	TTCGCCTAGCTGCAAAAGGA	337	PCR Reaction Mix (10 μL): Add 1.0 μL of 10× buffer, 0.2 μL of dNTPs, 0.2 μL each of forward and reverse primers, about 0.5 μL of DNA template, and 0.1 μL of Taq polymerase. Add nuclease-free water to reach a total of 10 μL. PCR Program: Start with 95°C for 5 minutes (initial denaturation), then run 25–35 cycles of: 95°C for 30 sec (denaturation), 55–60°C for 30 sec (annealing), and 72 °C for extension (adjust time based on fragment size). Finish with 72°C for 5–10 minutes, then hold at 4°C.
	F96-R1	CCAGAGGCAAGGCTATAGGC		
	F96-F2	TCGCCTAGCTGCACATGAAA	237	
	F96-R2	ACTGCCCTTCCCGTAGTAGT		
Forward Repeat F146	F146-F1	GGTTTCCCGATCGGAGATCC	398	
	F146-R1	GACTCTCCTACGGACCTGGT		
	F146-F2	CCCAGCGGAAAAGGCTAAGA	752	
	F146-R2	AGGGCCCCAAATGTCAATGT		
Palindromic Repeat P93	P93-F1	GGATCCTTCGCTGTTTGTGC	1013	
	P93-R1	AAGAGTACTTCGCGCCACAA		
	P93-F2	GCAGGGAGTGTGACAACGTA	1138	
	P93-R2	GATCCCGTCTCATTCCGACC		
Palindromic Repeat P135	P134-F1	CGCTAGATCCTGACCCCAAC	439	
	P134-R1	GCTCACTTCCCTCGTTCGAA		
	P134-F2	TTCAAGCCATAACACGCCCT	1029	
	P134-R2	TTCCATTCCTCGTGAGCCAC		
Palindromic Repeat P326	P326-F1	CGCTAGATCCTGACCCCAAC	593	
	P326-R1	AGAGGTAGCTACAGTCGCGA		
	P326-F2	AAAGGCCGCTGTCTCTTCTC	1169	
	P326-R2	AGACCACTCGACCGATCTCA		

The designation F1&R1:57 indicates that the annealing temperature for the primer pair F1&R1 was set at 57 degrees Celsius.

**Figure 4:**
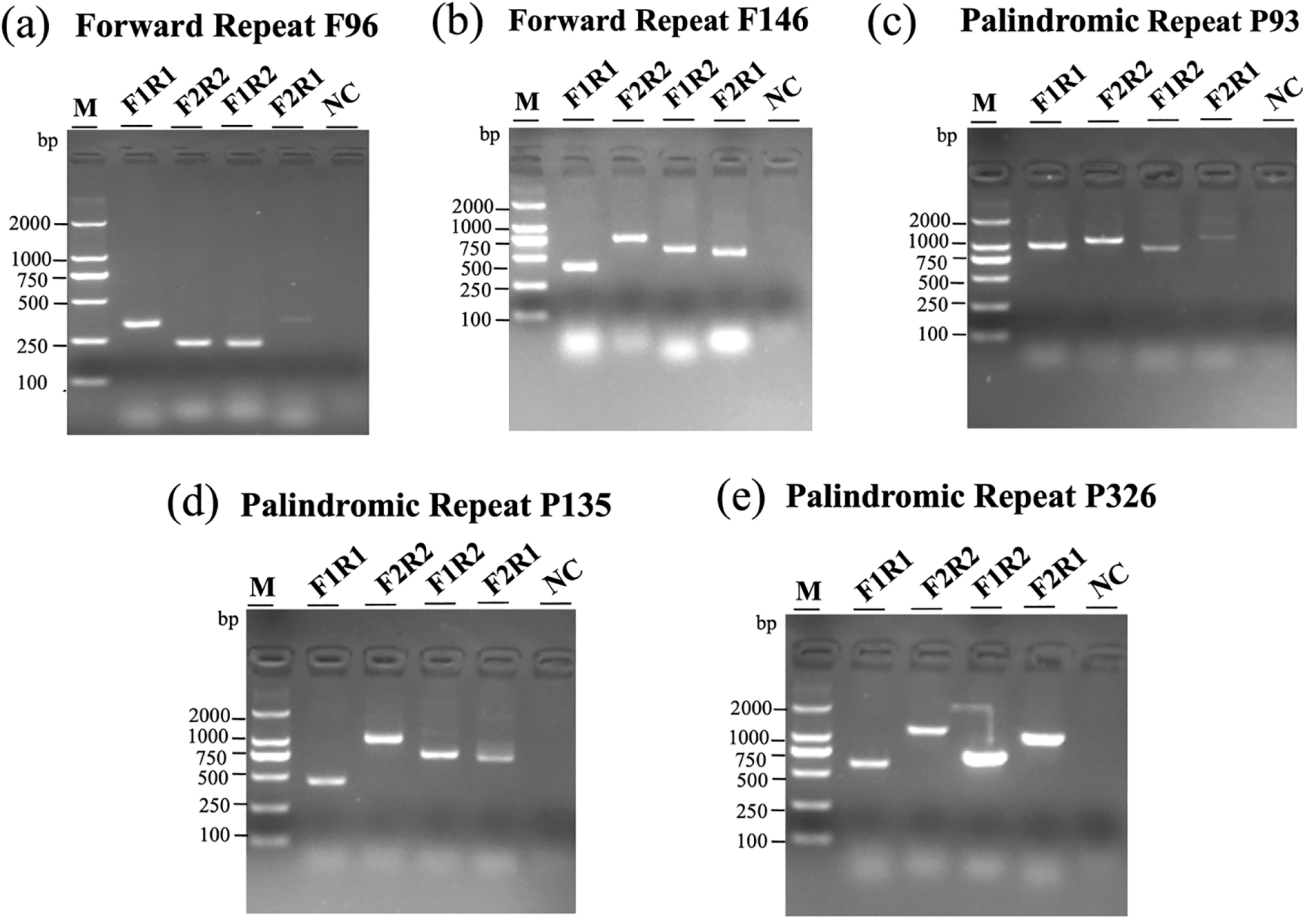
Structural variation of *I. indigotica* mitoDNA mediated by repeat recombination. **(a–e)** PCR amplification results showing multiple conformations of mitoDNA arising from recombination at two forward repeats (F96 and F146) and three palindromic repeats (P93, P134, and P326). Each gel includes: lane M, DNA marker; lanes F1R1 and F2R2, two major configurations; lanes F1R2 and F2R1, two minor configurations; NC, negative control. The observed band patterns reflect structural rearrangements driven by repeat-mediated recombination events.

As shown in **Figure 4**, agarose gel electrophoresis revealed distinct banding patterns for both native and recombinant configurations in multiple repeat pairs. For each repeat, at least two structural forms were detected: canonical conformations (F1R1 and F2R2) and recombined products (F1R2 and F2R1). The presence of clear bands in lanes corresponding to recombined primer pairs supports the hypothesis that repeat-mediated homologous recombination contributes to structural diversity in the *I. indigotica* mitogenome. Negative control lanes (NC) confirmed the specificity of the amplification reactions and excluded contamination. These findings highlight the dynamic nature of plant mitogenomes and the role of dispersed and palindromic repeats in generating structural variation.

### Structural features and comparative analysis of cpgenomes

The cpgenome of *I. indigotica* was successfully assembled with a total length of 153,821 bp and a typical quadripartite structure, comprising a large single-copy (LSC) region (83,577 bp), a small single-copy (SSC) region (17,702 bp), and two identical inverted repeat (IR) regions (26,271 bp each) (**Figure 2A**). Genome annotation identified gene clusters associated with photosystem I and II, ATP synthase, ribosomal proteins, and tRNAs, highlighting the conserved functionality of the cpgenome in photosynthesis and plastid gene expression. Functional categories were color-coded to distinguish among coding regions such as RNA polymerase subunits, NADH dehydrogenase components, and ribosomal RNA genes.

Comparative genome alignment revealed high conservation between the newly assembled cpgenome of *I. indigotica* and a previously reported sequence from the same species (OP620952), differing by only 6 base pairs in total length (**Figure 2B**). In contrast, alignment with the cpgenome of *I. tinctoria* (NC_028415) revealed minor sequence variations, suggesting evolutionary divergence between these congeneric species (**Figure 2C**). Further structural comparison of the LSC, SSC, and IR boundary regions between *I. indigotica* and *I. tinctoria* uncovered shifts in the positions of junctions such as JLB, JSB, JSA, and JLA, along with differences in adjacent gene lengths and arrangements (**Figure 2D**). These structural differences at IR boundaries, including variable placement of genes such as *ycf1*, *rps19*, and *ndhF*, underscore the dynamic nature of cpgenome evolution within the *Isatis* genus.

### Organellar and nuclear integration of mitochondrial sequences

**Figure 5:**
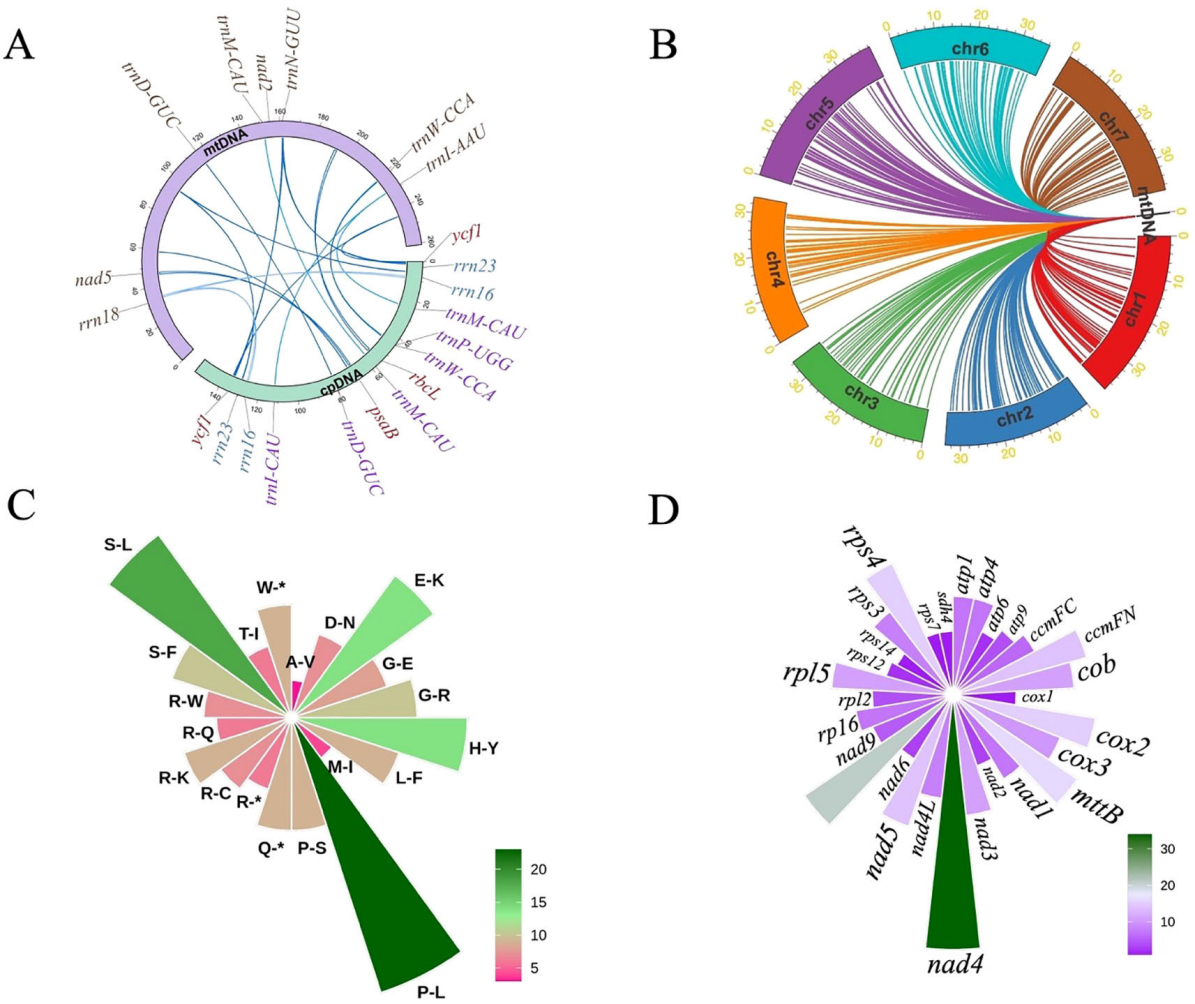
Analysis of sequence transfer, RNA editing, and gene interactions in the mitogenome of *I. indigotica.* **(A)** Sequence transfer between the mtDNA and cpDNA of *I. indigotica*, with transferred genes and regions indicated. Blue lines represent homologous fragments. **(B)** Integration of mitoDNA fragments into the nuclear genome of *I. indigotica*, based on reference genome data from NCBI (accession number VHIU00000000). Colored lines link mitochondrial sequences to corresponding locations on nuclear chromosomes chr1–chr7. **(C)** Predicted RNA editing events in *I. indigotica* mitochondrial genes, represented as amino acid conversions. The color scale indicates the frequency of each type of editing event. **(D)** Distribution of RNA editing events across mitochondrial genes, with *nad4* showing the highest number of editing sites. Bar lengths indicate the editing frequency per gene.

Intracellular DNA transfer is a common feature of plant genomes, contributing to genomic complexity and evolutionary adaptation. In the mitogenome of *I. indigotica*, a total of 17 mitochondrial plastid DNA transfers (MTPTs) were identified, with sequence lengths ranging from 74 bp to 1,367 bp and sequence identity between 74.4% and 99.8% (**Supplementary Table S6**). These MTPTs collectively span several kilobases and reflect frequent plastid-to-mitochondrion DNA movement (**Figure 5A**). Among them, several MTPTs contained fragments of PCGs, such as *rbcL* (MTPT1), *psaB* (MTPT2, MTPT11), and *ycf1* (MTPT3, MTPT4, MTPT9, MTPT10), indicating historical transfers of functional genetic elements. Notably, MTPT3 and MTPT4 represent bidirectional transfers of the same *ycf1* fragment, with nearly complete sequence identity (99.8%) and reverse orientation.

In addition to PCGs, various ribosomal RNA and tRNA genes were also identified among MTPTs. For example, MTPT5 and MTPT6 encode partial sequences of *rrn23*, while MTPT7 and MTPT8 harbor homologs of *rrn16*, though with lower identity (~74%) and higher mismatch counts, suggesting older or less conserved transfers. tRNA-related MTPTs include *trnP-UGG* (MTPT12), *trnW-CCA* (MTPT13), *trnD-GUC* (MTPT14), and *trnM-CAU* (MTPT15, MTPT16), as well as *trnI-CAU* (MTPT17), showing varying levels of conservation. These findings indicate that not only protein-coding but also structural RNA elements were subject to intracellular translocation. While some tRNAs may retain functional integrity, others, particularly those with high mismatch and gap counts, may be relics of ancient transfers with limited biological function.

Beyond organelle–organelle transfer, comparative analysis based on the *I. indigotica* reference nuclear genome (NCBI accession VHIU00000000) revealed extensive integration of mitochondrial DNA (NUMTs) into the nuclear genome. As illustrated in **Figure 5B**, mitochondrial fragments were mapped to all seven nuclear chromosomes (chr1–chr7), with chr1, chr2, and chr5 exhibiting particularly dense insertion patterns. These NUMTs contribute to nuclear genome expansion and serve as evolutionary footprints of organellar-nuclear interaction. Together, the presence of MTPTs and NUMTs highlights the dynamic and ongoing exchange of genetic material between organellar and nuclear compartments in *I. indigotica*, providing important insights into organellar genome evolution and intracellular genomic plasticity.

### Consistent patterns of RNA editing in PCGs revealed by comparative prediction and transcriptome-based analyses

The prediction-based strategy identified 380 putative C-to-U RNA-editing sites across mitochondrial PCGs of *I.indigotica*, based on conserved editing patterns from related species using PREPACT3 (**Supplementary Table S7**). These predicted edits predominantly resulted in nonsynonymous changes, with a strong preference for specific amino acid substitutions. Notably, conversions from serine (S) to leucine (L), proline (P) to leucine (L), and serine (S) to phenylalanine (F) were the most frequent, suggesting potential functional shifts in hydrophobicity and structural properties of mitochondrial proteins. Gene-wise analysis revealed that *nad4*, *ccmFn*, and *ccmB* harbored the greatest number of editing sites, indicating that genes involved in respiratory and cytochrome c biogenesis processes are frequent targets of RNA editing (**Supplementary Figure S2**). A detailed classification of RNA-editing events based on amino acid property transitions (**Supplementary Table S8**) further highlights these tendencies. Of the 357 identified nonsynonymous edits, nearly half (48.74%) were transitions from hydrophilic to hydrophobic residues, indicating a substantial shift toward increased protein hydrophobicity. This group was dominated by conversions such as TCA (S) → TTA (L), TCT (S) → TTT (F), and CGG (R) → TGG (W). Hydrophobic-to-hydrophobic transitions accounted for 28.29%, mainly due to P → L and A → V substitutions. Hydrophilic-to-hydrophilic and hydrophobic-to-hydrophilic edits comprised 14.00% and 8.40%, respectively, with changes like CGT (R) → TGT (C) and CCT (P) → TCT (S) being prominent. Additionally, two editing events (0.56%) led to stop codons, potentially serving regulatory or degradation-related roles. This distribution emphasizes the functional importance of RNA editing in refining the hydrophobicity and polarity of mitochondrial proteins, thereby influencing their structural and biochemical dynamics.

In parallel, transcriptome read mapping against the *I. indigotica* mitogenome (C_AA108663) identified 230 RNA-editing sites (**Figures 5C, D**), derived from variant calling of publicly available RNA-seq data. Despite a lower total count, the editing spectrum overlapped substantially with prediction results. Key substitutions such as S→L, P→L, and S→F were consistently observed, and *nad4* remained the most edited gene. These findings indicate that high-confidence editing events are reproducible across methodologies, and that a conserved subset of edits is likely to be biologically relevant. The concordant amino acid change patterns support the hypothesis that RNA editing modulates protein properties in a directionally selective manner, favoring alterations in polarity and hydrophobicity to maintain or optimize protein function within the mitochondrial environment.

### Mitogenome evolution and comparative genomics

**Figure 6:**
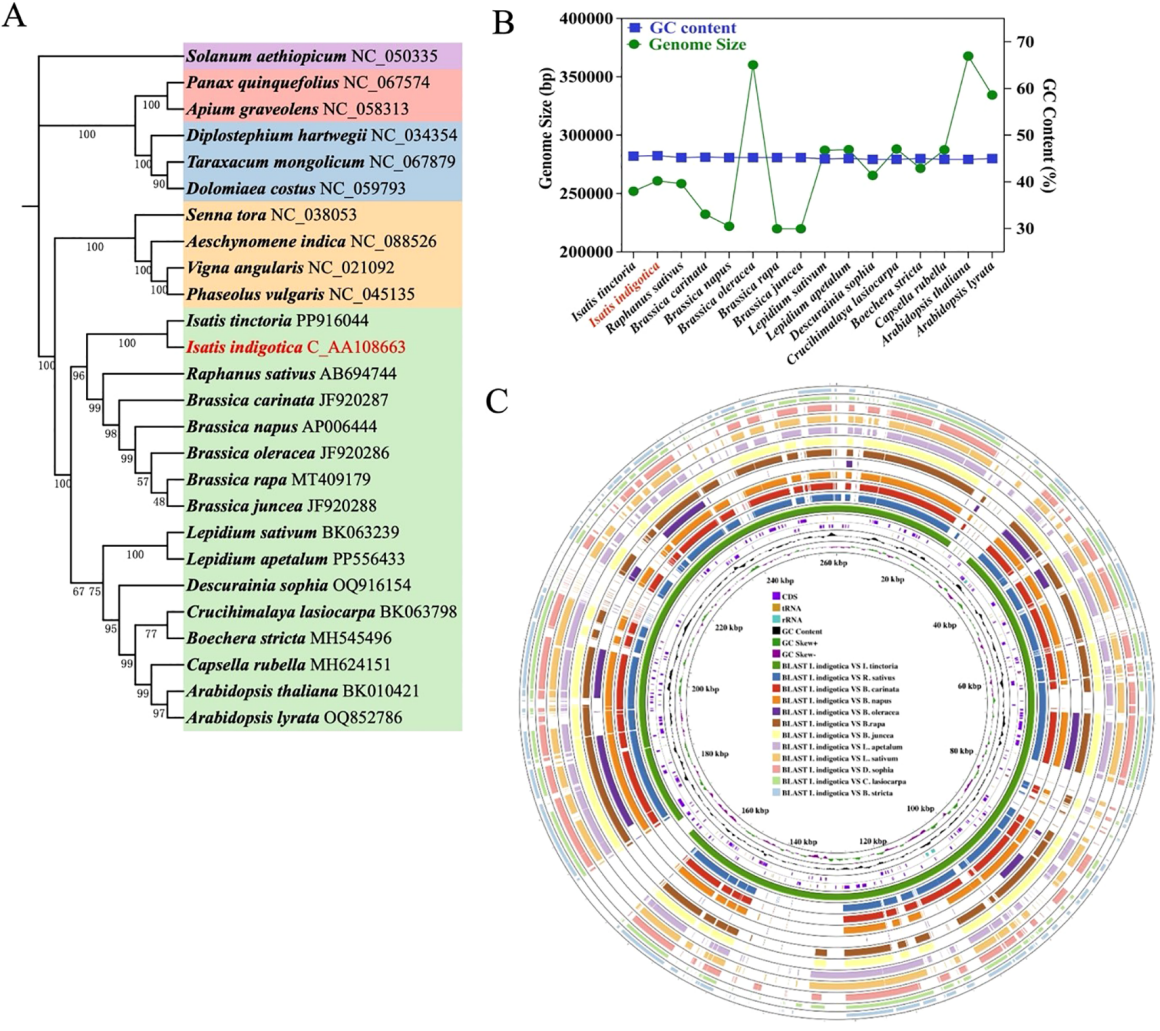
Mitogenome evolution and comparative analysis of *I. indigotica.* **(A)** Phylogenetic tree of 26 plant species based on conserved mitochondrial PCGs, highlighting the position of *I. indigotica* (in red) and its evolutionary relationships within the Brassicales and related taxa. **(B)** Comparison of mitogenome size and GC content across 18 Brassicaceae species, with *I. indigotica* marked in red. **(C)** Circular representation of the *I. indigotica* mitogenome, showing annotated features including CDS, tRNA, rRNA, GC content, and GC skew. The outer rings display BLAST comparisons between *I. indigotica* and 12 other species, revealing conserved regions and structural variations.

Based on **Figure 6**, the evolutionary and structural characteristics of the *I. indigotica* mitogenome were investigated through phylogenetic analysis, genome feature comparison, and multi-species alignment. The phylogenetic tree (**Figure 6A**), constructed from conserved mitochondrial PCGs across 26 representative angiosperm species, demonstrates that *I. indigotica* clusters tightly with *I. tinctoria*, confirming their close genetic relationship within the *Brassicaceae* family. Other genera, such as *Raphanus*, *Brassica*, and *Lepidium*, also form distinct, well-supported clades, reflecting the broader phylogenetic framework within the order Brassicales. The high bootstrap values across the tree nodes provide robust support for the inferred relationships and indicate strong conservation among mitochondrial genes across these taxa. This phylogenetic placement underscores the evolutionary consistency in mitogenomes within *Brassicaceae* and affirms the use of mitochondrial PCGs as reliable markers for resolving interspecies relationships. Furthermore, the comparison of mitogenome size and GC content among 18 *Brassicaceae* species (**Figure 6B**) reveals that although the total mitogenome size varies widely, ranging from approximately 220,000 to over 390,000 base pairs, the GC content remains relatively stable across species, typically clustering between 44% and 46%. *Isatis tinctoria*, marked in red, exhibits a genome size of approximately 283 kb with a GC content of ~45%, placing it near the average within its family. This trend suggests that while genome size can undergo lineage-specific expansion or reduction due to structural rearrangements or integration of foreign sequences, the GC content is more evolutionarily conserved and may be subject to functional constraints related to DNA stability or transcription efficiency.

In addition to phylogenetic and compositional features, the circular genome visualization (**Figure 6C**) offers a comprehensive view of the *I. indigotica* mitogenome and its structural architecture. The annotated internal rings display key genomic components, including coding sequences (CDS), transfer RNAs (tRNAs), ribosomal RNAs (rRNAs), GC content, and GC skew, which collectively reflect the functional landscape of the mitogenome. Surrounding these are twelve concentric outer rings that depict BLAST-based alignments between *I. indigotica* and twelve other selected species within *Brassicaceae*, enabling a comparative analysis of genome conservation and divergence. The presence of numerous conserved syntenic blocks indicates that many mitochondrial regions—particularly those associated with essential genes such as *cox*, *nad*, *atp*, and *ccm*—are strongly preserved across species, consistent with their critical roles in mitochondrial respiration and metabolism. At the same time, breaks and shifts in alignment patterns highlight lineage-specific rearrangements and structural variation, which may result from recombination, gene loss, or horizontal gene transfer events. These structural differences, although variable, often spare the core mitochondrial gene set, reinforcing the notion that while non-coding and intergenic regions are more evolutionarily plastic, the coding content remains stable to ensure essential mitochondrial functions. Taken together, this integrated analysis affirms that *I. indigotica* possesses a structurally stable mitogenome that shares extensive sequence similarity with its close relatives, while also harboring unique features that may reflect its individual evolutionary history. These findings provide valuable insight into mitogenome evolution within *Brassicaceae* and set a foundation for future investigations into mitochondrial gene function, inheritance, and adaptation.

### Syntenic conservation and structural rearrangement in plant mitogenomes

**Figure 7:**
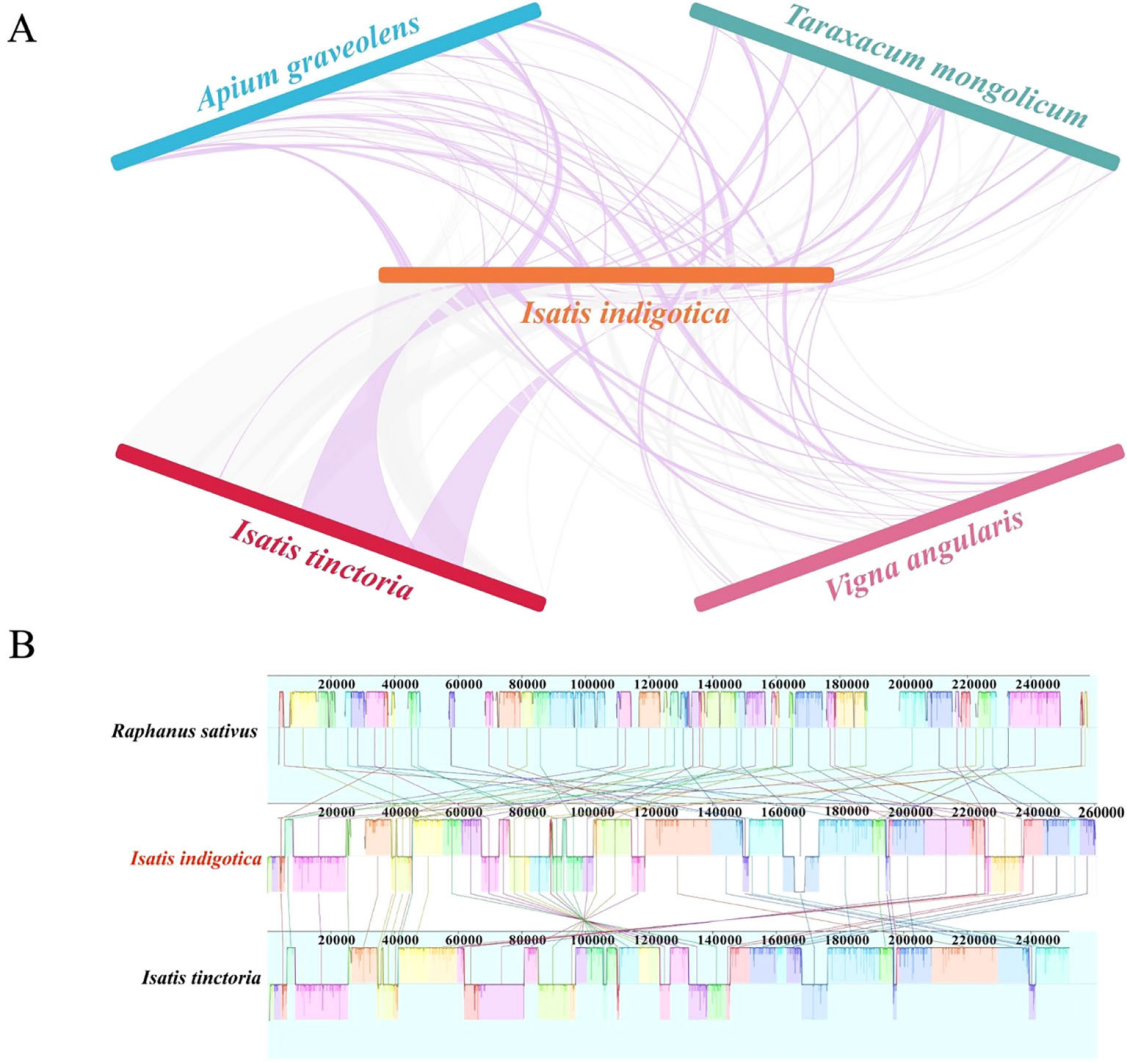
Synteny and collinearity analysis of the *I. indigotica* mitogenome. **(A)** Synteny relationships between *I. indigotica* and four other species (*I. tinctoria*, *A. graveolens*, *T. mongolicum*, and *V. angularis*), showing shared homologous regions and genome rearrangements. **(B)** Collinearity comparison among the mitogenomes of *I. indigotica*, . ***I*** *tinctoria*, and *R. sativus*, revealing conserved blocks and structural variations across the genomes.

The synteny and collinearity analyses of the mitogenome reveal notable patterns of both conservation and structural reconfiguration across different plant species. The syntenic comparisons, as illustrated in **Figure 7A**, show that extensive homologous sequence regions are retained between the focal genome and those of four other species: *I. tinctoria*, *A. graveolens*, *T. mongolicum*, and *V. angularis* (**Supplementary Figure S9**). These homologous segments are represented by connecting curves, varying in intensity and continuity, which suggest differing degrees of sequence similarity and genome structural stability. The greatest density and continuity of these curves are observed between the focal genome and *I. tinctoria*, indicating a high degree of conservation in sequence composition and segmental arrangement. By contrast, the connections with the other three species appear more sporadic and fragmented, suggesting a comparatively lower level of sequence conservation and a higher degree of rearrangement. This discrepancy likely reflects differing evolutionary distances, with more distantly related species exhibiting greater divergence in mitogenome architecture.

Further insights into structural organization are provided by the collinearity analysis in **Figure 7B**, which focuses on three species: *R. sativus*, *I. tinctoria*, and the focal taxon. The visualization of LCBs, each demarcated by uniquely colored segments, facilitates a direct comparison of gene order, orientation, and overall genomic architecture. A pronounced similarity in block arrangement and orientation is evident between the focal genome and *I. tinctoria*, reinforcing the observation of strong genomic conservation inferred from the synteny analysis. In contrast, the alignment with *R. sativus* reveals a more disrupted block structure, characterized by numerous inversions, translocations, and possible insertions or deletions. These structural rearrangements result in a visually more complex and less orderly pattern of alignment, reflecting a greater divergence in genome structure. The differential collinearity among these three species suggests that mitogenome evolution in plants involves a balance between the retention of conserved functional elements and lineage-specific rearrangements. Such rearrangements may occur through recombination, duplication, or other mechanisms of genome plasticity, which contribute to the observed diversity in mitogenome organization across plant lineages.

### Divergent evolutionary patterns of PCGs among angiosperms

To investigate the evolutionary dynamics of PCGs among angiosperms, three complementary analyses were conducted: gene copy number variation, Ka/Ks ratios, and Pi. Together, these results elucidate the extent of functional conservation, evolutionary constraint, and sequence variability across diverse lineages, with particular attention to the Brassicales and Fabales clades.

**Figure 8:**
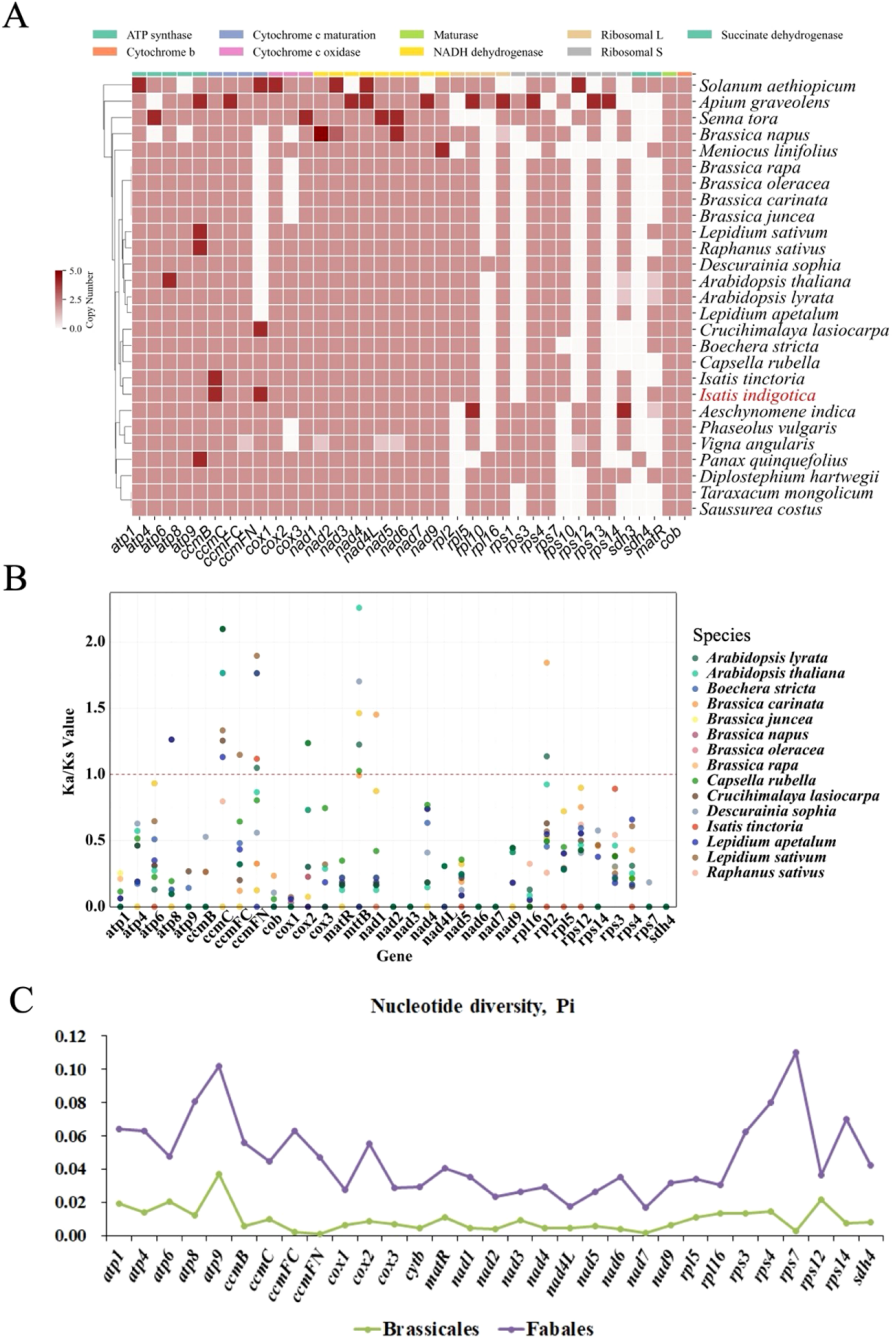
Comparative genomic and evolutionary analysis of mitochondrial genes in *I. indigotica* and related species. **(A)** Heatmap of mitochondrial PCGs copy numbers across 30 plant species, with functional categories indicated by color. The distribution reveals variation in gene retention and duplication among species, including *I. indigotica* (red). **(B)** Pairwise Ka/Ks ratios for shared mitochondrial genes among 17 Brassicaceae species, indicating patterns of selective pressure acting on individual genes. **(C)** Pi of mitochondrial genes between *Brassicales* and *Fabales* species, showing genetic variability within each group.

Gene copy number profiling (**Figure 8A**) revealed a heterogeneous landscape across the surveyed taxa. While the majority of PCGs were retained as single-copy genes in most species—particularly those involved in essential energy metabolism pathways such as ATP synthesis (e.g., *atp1*, *atp6*, *atp9*) and cytochrome complexes (e.g., *cob, cox1–3*)—a subset of genes exhibited lineage-specific duplications or absences. For instance, duplications in genes such as *rps12*, *nad4L*, and *ccmFN* were observed in several *Brassicaceae* members, suggesting possible subfunctionalization or compensatory mechanisms in mitochondrial translation and redox regulation. Meanwhile, frequent losses or underrepresentation of ribosomal protein genes (e.g., *rpl2*, *rps1*, *rps14*) in multiple lineages may indicate ongoing nuclear transfer or pseudogenization events. These patterns reflect both the functional rigidity and evolutionary plasticity of plant mitogenomes, where essential bioenergetic functions are maintained under strict constraint, while other genes tolerate structural fluidity.

The evolutionary constraints acting upon these genes were further evaluated through Ka/Ks analysis (**Figure 8B**), focusing on 16 representative *Brassicaceae* species. As expected for mitogenomes, purifying selection dominated across most PCGs, with Ka/Ks ratios predominantly below 1.0. However, exceptions were noted in genes such as ccmB, matR, and rps3, where elevated Ka/Ks values in specific taxa suggested relaxed selection or episodic positive selection. Notably, the variability observed in cytochrome c maturation genes (*ccmB*, *ccmC*, *ccmFN*) and ribosomal proteins (*rps3*, *rps4*) implies that these loci may be subject to adaptive pressures, potentially in response to environmental cues or cytonuclear co-evolution. Such divergence highlights regions of the mitogenome that may play a disproportionate role in lineage-specific adaptation or reproductive isolation.

Complementary to these findings, Pi analysis (**Figure 8C**) offered a broader population-level perspective on genetic variability. A clear disparity emerged between Brassicales and Fabales, with the latter exhibiting consistently higher Pi values across nearly all mitochondrial genes. Genes such as *rps12*, *nad6*, and *ccmFC* in Fabales showed particularly elevated polymorphism, indicating more rapid sequence turnover or relaxed constraints within this order. In contrast, Brassicales maintained low Pi, underscoring the genomic stability and strong conservation of mitogenomic content in this lineage. This contrast between lineages not only mirrors their differing evolutionary histories but also supports the notion that mitogenomes, though structurally conserved, can exhibit considerable heterogeneity in their molecular evolution.

## Discussion

The complete mitochondrial and cpgenome assemblies presented in this study provide valuable genetic resources for investigating the organellar biology and evolutionary history of *I. indigotica*. By utilizing high-fidelity long-read sequencing and implementing integrative annotation strategies, this research addresses the complexity of organelle genome structure, particularly the challenges associated with extensive repeat regions and recombination events characteristic of plant mitogenomes. The circular-mapping mitogenome of *I. indigotica* spans 260,864 bp and contains 65 unique genes, including all 23 core mitochondrial PCGs, consistent with gene content observed in other *Brassicaceae* species.

A notable feature of the mitogenome is the abundance and diversity of repeat elements, including SSRs, tandem repeats, and dispersed repeats. These repetitive elements, especially the medium-to-large dispersed repeats, were shown to mediate recombination events, as evidenced by the presence of multiple structural isoforms validated through both in silico prediction and experimental PCR confirmation. This dynamic recombination landscape suggests that *I. indigotica* maintains a multipartite mitogenome architecture, in line with other angiosperms exhibiting structural plasticity.

From a taxonomic perspective, the distinction between *I. indigotica* and *I. tinctoria* has been a subject of ongoing debate. Although both species belong to the same genus and exhibit considerable morphological and genetic similarity, their practical applications and ethnobotanical roles diverge significantly. *Isatis indigotica* is predominantly used in traditional Chinese medicine for its antiviral and anti-inflammatory properties, whereas *I. tinctoria* has historically been cultivated as a source of indigo dye in Europe. This study offers the first comprehensive organellar genomic comparison between the two species based on cell line-derived mitochondrial sequences. The observed synteny and local rearrangements provide molecular evidence of their close genetic relationship, while also revealing distinct structural variations that may be linked to lineage-specific functional adaptation or domestication history.

RNA editing plays a critical role in the post-transcriptional modification of mitochondrial transcripts. A total of 587 RNA editing sites were predicted within PCGs, primarily involving C-to-U conversions. Most edits result in nonsynonymous changes, favoring the substitution of hydrophilic residues with hydrophobic ones, which may affect protein stability or membrane integration. In this study, two complementary strategies were employed to assess RNA editing patterns: computational prediction and transcriptome-based empirical validation. While prediction tools such as PREPACT revealed the full landscape of putative editing events, mapping of RNA-seq reads allowed for the confirmation of 380 high-confidence sites. The convergence between predicted and transcript-supported editing patterns—particularly in genes such as *nad4*, *ccmFn*, and *ccmB*—provides strong evidence that these modifications are functionally relevant. The dual-method approach enhances the robustness of RNA editing detection and helps to reconcile discrepancies often encountered in single-method studies.

Comparative analysis of mitogenomes further supports the close phylogenetic relationship between *I. indigotica* and *Raphanus sativus*, as initially suggested by chloroplast phylogenies (Yang and Wang, 2017). Mitogenomic comparisons reveal notable synteny and structural correspondence between these two species, characterized by conserved gene content and locally aligned genomic blocks. However, despite this broad conservation, lineage-specific rearrangements—including inversions and translocations—are evident, highlighting the dynamic nature of mitogenome evolution. Whole-genome dot plot analyses and synteny mapping underscore this pattern: while syntenic regions are often disrupted across distantly related taxa, a more continuous alignment is observed between *I. indigotica* and other *Brassicaceae* members, particularly *R. sativus*. These structural similarities lend further support to their sister relationship and suggest that mitogenomic architecture, in addition to gene content, can serve as an informative phylogenetic signal within the family.

Phylogenetic reconstruction based on 28 conserved mitochondrial PCGs placed *I. indigotica* firmly within the *Brassicaceae* clade, closely aligned with other *Cruciferae* species. Genome size and GC content across examined mitogenomes show moderate variation, with *I. indigotica* exhibiting values well within the expected range for its taxonomic group. The presence of MTPTs and mitochondrial nuclear DNA insertions highlights ongoing intracellular DNA transfer, suggesting a dynamic genomic interplay between organelles and the nucleus. The integration of plastid and mitochondrial sequences, particularly involving tRNA and rRNA gene fragments, may contribute to genome expansion and functional redundancy.

In summary, the structural, functional, and comparative genomic analyses of the *I. indigotica* mitogenome provide significant insights into plant mitogenome evolution, with emphasis on repeat-mediated recombination, RNA editing, and inter-organellar DNA transfer. The dual approach to RNA editing analysis and the focused comparison with *I. tinctoria* also address existing gaps in taxonomic clarification and transcriptomic post-processing in *Brassicaceae*. These findings offer a robust foundation for further evolutionary, physiological, and pharmacological studies in this important medicinal lineage.

## Conclusion

This study provides new insights into the mitochondrial genome of *I. indigotica*, shedding light on its structure, function, and evolutionary characteristics. The complete mitogenome, consisting of 260,864 base pairs, includes 31 protein-coding genes, 21 transfer RNAs, and 3 ribosomal RNAs, along with notable repeat-mediated recombination events. These features suggest active genome rearrangements and stability essential for mitochondrial function. The study also emphasizes the importance of RNA editing, particularly C-to-U conversions, in stabilizing and optimizing mitochondrial proteins critical for respiration and cytochrome c biogenesis. Additionally, phylogenetic analysis with other Brassicaceae species revealed a close relationship between *I. indigotica* and Brassica species, highlighting the ongoing genetic exchange between mitochondria and the nucleus, particularly through mitochondrial-nuclear DNA integrations. Overall, the findings contribute significantly to our understanding of the dynamic interaction between nuclear and organellar genomes, offering valuable perspectives for future research on plant mitochondrial evolution and its relevance to medicinal plant studies.

## Data Availability

The sequencing data produced in this project have been archived in the Genome Sequence Archive (GSA) at the National Genomics Data Center (NGDC), accessible under accession number CRA026873 (https://ngdc.cncb.ac.cn/gsa) (Members and Partners, 2025). In addition, corresponding annotated genome information has been submitted to GenBase at NGDC/CNCB and is available under accession number C_AA108663.1 (https://ngdc.cncb.ac.cn/genbase) (Bu et al., 2024).

## Glossary

MTPTs: mitochondrial plastid DNAs
NUMTs: nuclear mitochondrial DNAs
cpDNA: chloroplast DNA
PCGs: protein-coding genes
SSRs: simple sequence repeats
tRNAs: transfer RNAs
rRNAs: ribosomal RNAs
RNA: ribonucleic acid
RSCU: relative synonymous codon usage
Ka/Ks: nonsynonymous/synonymous substitution ratio
Pi: nucleotide diversity
MSA: multiple sequence alignment
CDS: coding sequence
GFF3: General Feature Format version 3
GC: guanine–cytosine content
ATP: adenosine triphosphate
NADH: nicotinamide adenine dinucleotide hydride
ccm: cytochrome c biogenesis genes
cox: cytochrome c oxidase genes
cob: cytochrome b gene
matR: maturase-related gene
LSC: large single-copy
SSC: small single-copy
IR: inverted repeat
N50: minimum contig length covering 50% of the genome
mt: mitochondrial contig
APG: Angiosperm Phylogeny Group
BLAST: Basic Local Alignment Search Tool
PCR: polymerase chain reaction
CTAB: cetyltrimethylammonium bromide
DEPC: diethyl pyrocarbonate
Qubit: fluorometric DNA quantification system
NanoDrop: UV-based spectrophotometer
Leu: leucine
Val: valine
Ser: serine
Arg: arginine
Gly: glycine.
