# Supplementary material for: Mitochondrial genome of Isatis indigotica reveals repeat-mediated recombination and phylogenetic insights in Cruciferae
Source: Front Plant Sci. 2025 Oct 15;16:1655810. doi: 10.3389/fpls.2025.1655810 (PMC12568568; doi:10.3389/fpls.2025.1655810)
Supplement: Supplementary file 1 [file Table1.docx]

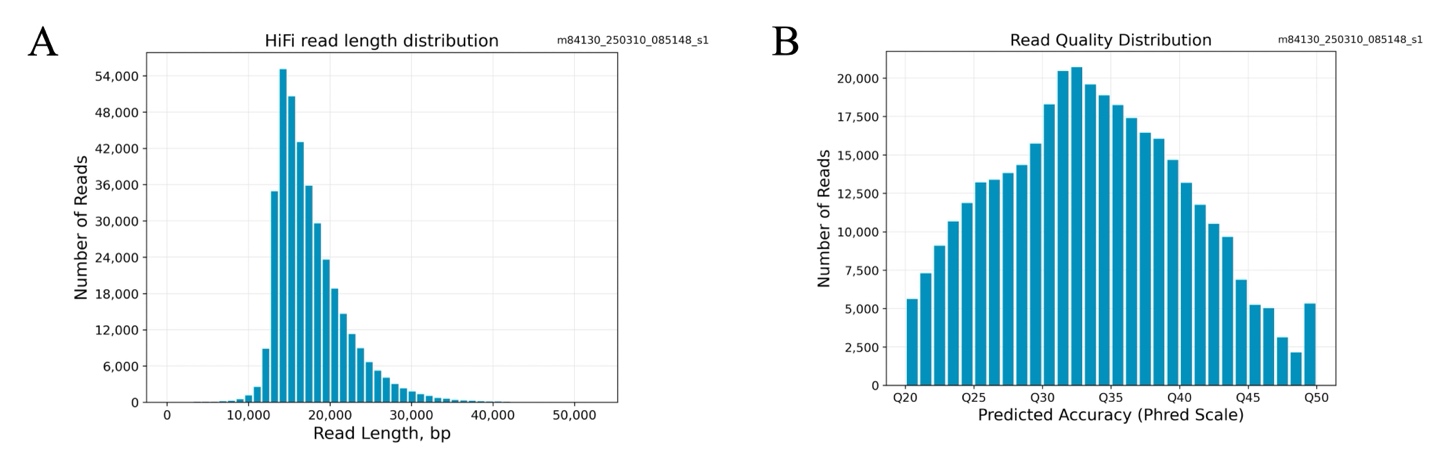
**Figure S1 | HiFi Read Statistics for I. indigotica Mitogenome Sequencing**. **(A)** Distribution of HiFi read lengths. **(B)** Distribution of predicted read quality (Q score) across HiFi reads.
