# Supplementary material for: Mitochondrial genome of Isatis indigotica reveals repeat-mediated recombination and phylogenetic insights in Cruciferae
Source: Front Plant Sci. 2025 Oct 15;16:1655810. doi: 10.3389/fpls.2025.1655810 (PMC12568568; doi:10.3389/fpls.2025.1655810)

**Figure S2 | Predicted Distribution of RNA Editing Events by Amino Acid Substitution and Gene in the Mitogenome**. (A) Frequency of predicted amino acid substitutions resulting from C-to-U RNA editing, based on PREPACT3 analysis. Dominant transitions include S→L, P→L, and S→F, indicating strong preferences in editing patterns. (B) Gene-wise distribution of predicted RNA editing events, showing that nad4, ccmB, and ccmFn harbor the highest number of editing sites, particularly among genes involved in respiratory function and cytochrome c biogenesis.


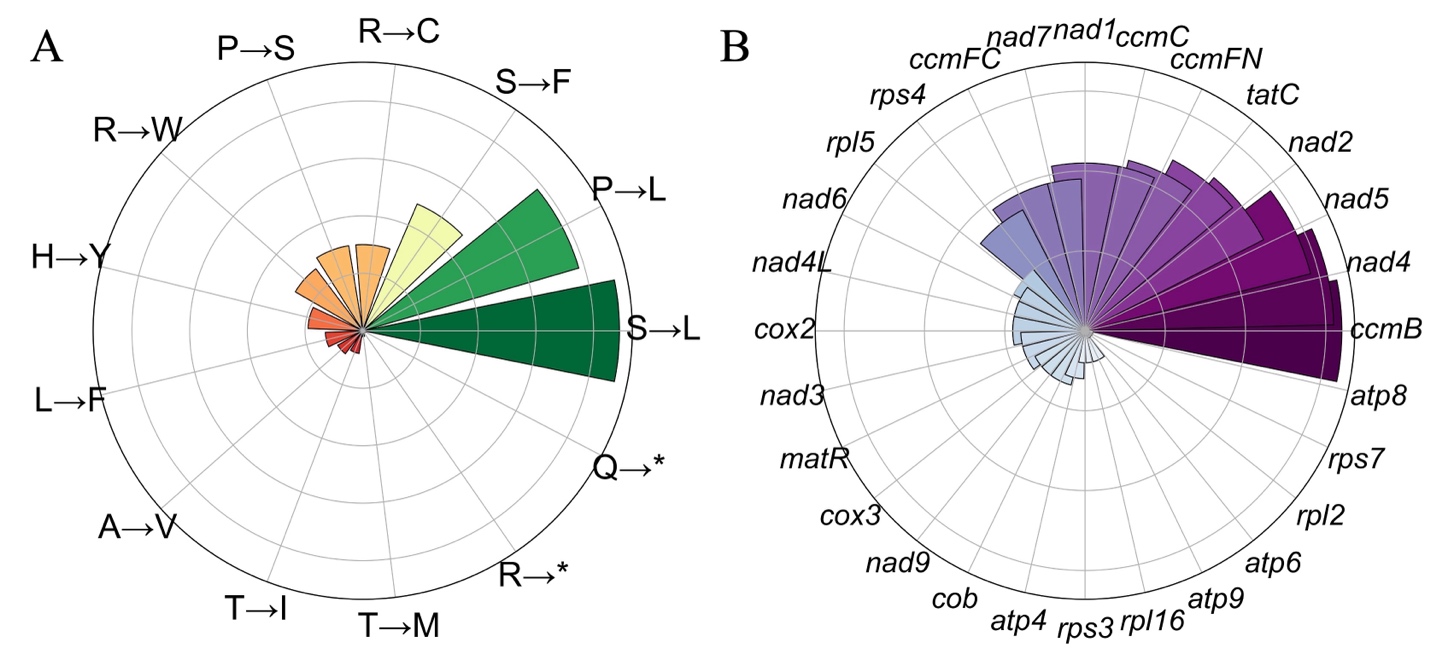

Supplement: Supplementary file 2 [file Table2.docx]
