# Supplementary material for: Mitochondrial genome of Isatis indigotica reveals repeat-mediated recombination and phylogenetic insights in Cruciferae
Source: Front Plant Sci. 2025 Oct 15;16:1655810. doi: 10.3389/fpls.2025.1655810 (PMC12568568; doi:10.3389/fpls.2025.1655810)
Supplement: Supplementary file 3 [file Table3.docx]

**Table S1 | Comparative Dataset of Mitogenomes Across Angiosperms Including Taxonomic Classification, Genome Size, and AT Content**

| **NCBI Accession NO.** | **Organism** | **Class** | **Order** | **Superfamily** | **Family** | **Genus** | **Length (bp)** | **AT%** |
| --- | --- | --- | --- | --- | --- | --- | --- | --- |
| NC_088526.1 | Aeschynomene indica | Magnoliopsida | Fabales | Magnoliopsida | Fabaceae | Aeschynomene | 388249 | 54.9 |
| NC_058313.1 | Apium graveolens | Magnoliopsida | Apiales | Magnoliopsida | Apiaceae | Apium | 371275 | 54.9 |
| OQ852786.1 | Arabidopsis lyrata | Magnoliopsida | Brassicales | Magnoliopsida | Brassicaceae | Arabidopsis | 334431 | 55 |
| BK010421.1 | Arabidopsis thaliana | Magnoliopsida | Brassicales | Magnoliopsida | Brassicaceae | Arabidopsis | 367808 | 55.2 |
| MH545496.1 | Boechera stricta | Magnoliopsida | Brassicales | Magnoliopsida | Brassicaceae | Boechera | 271601 | 55 |
| JF920287.1 | Brassica carinata | Magnoliopsida | Brassicales | Magnoliopsida | Brassicaceae | Brassica | 232241 | 54.7 |
| JF920288.1 | Brassica juncea | Magnoliopsida | Brassicales | Magnoliopsida | Brassicaceae | Brassica | 219766 | 54.8 |
| AP006444.1 | Brassica napus | Magnoliopsida | Brassicales | Magnoliopsida | Brassicaceae | Brassica | 221853 | 54.8 |
| JF920286.1 | Brassica oleracea | Magnoliopsida | Brassicales | Magnoliopsida | Brassicaceae | Brassica | 360271 | 54.8 |
| MT409179.1 | Brassica rapa | Magnoliopsida | Brassicales | Magnoliopsida | Brassicaceae | Brassica | 219736 | 54.8 |
| MH624151.1 | Capsella rubella | Magnoliopsida | Brassicales | Magnoliopsida | Brassicaceae | Capsella | 287405 | 55.2 |
| BK063798.1 | Crucihimalaya lasiocarpa | Magnoliopsida | Brassicales | Magnoliopsida | Brassicaceae | Crucihimalaya | 288122 | 55.2 |
| OQ916154.1 | Descurainia sophia | Magnoliopsida | Brassicales | Magnoliopsida | Brassicaceae | Descurainia | 265457 | 55.2 |
| NC_034354.1 | Diplostephium hartwegii | Magnoliopsida | Asterales | Magnoliopsida | Asteraceae | Diplostephium | 277718 | 55.1 |
| NC_059793.1 | Saussurea costus | Magnoliopsida | Asterales | Magnoliopsida | Asteraceae | Dolomiaea | 320439 | 55 |
| C_AA108663.1 | Isatis indigotica | Magnoliopsida | Brassicales | Magnoliopsida | Brassicaceae | Isatis | 260864 | 54.4 |
| PP916044.1 | Isatis tinctoria | Magnoliopsida | Brassicales | Magnoliopsida | Brassicaceae | Isatis | 251922 | 54.5 |
| PP556433.1 | Lepidium apetalum | Magnoliopsida | Brassicales | Magnoliopsida | Brassicaceae | Lepidium | 287626 | 55 |
| BK063239.1 | Lepidium sativum | Magnoliopsida | Brassicales | Magnoliopsida | Brassicaceae | Lepidium | 287054 | 55.1 |
| NC_067574.1 | Panax quinquefolius | Magnoliopsida | Apiales | Magnoliopsida | Araliaceae | Panax | 573154 | 55.3 |
| NC_045135.1 | Phaseolus vulgaris | Magnoliopsida | Fabales | Magnoliopsida | Fabaceae | Phaseolus | 395516 | 54.9 |
| AB694744.1 | Raphanus sativus | Magnoliopsida | Brassicales | Magnoliopsida | Brassicaceae | Raphanus | 258426 | 54.8 |
| NC_038053.1 | Senna tora | Magnoliopsida | Fabales | Magnoliopsida | Fabaceae | Senna | 566589 | 54.8 |
| NC_050335.1 | Solanum aethiopicum | Magnoliopsida | Solanales | Magnoliopsida | Solanaceae | Solanum | 566695 | 55.3 |
| NC_067879.1 | Taraxacum mongolicum | Magnoliopsida | Asterales | Magnoliopsida | Asteraceae | Taraxacum | 304467 | 54.5 |
| NC_021092.1 | Vigna angularis | Magnoliopsida | Fabales | Magnoliopsida | Fabaceae | Vigna | 404466 | 54.8 |
