# Supplementary material for: Mitochondrial genome of Isatis indigotica reveals repeat-mediated recombination and phylogenetic insights in Cruciferae
Source: Front Plant Sci. 2025 Oct 15;16:1655810. doi: 10.3389/fpls.2025.1655810 (PMC12568568; doi:10.3389/fpls.2025.1655810)
Supplement: Supplementary file 4 [file Table4.docx]

**Table S2 | RSCU Values for Mitochondrial PCGs.**

| **Amino** | **Codon 1 RSCU** | **Codon 2 RSCU** | **Codon 3 RSCU** | **Codon 4 RSCU** | **Codon 5 RSCU** | **Codon 6 RSCU** |
| --- | --- | --- | --- | --- | --- | --- |
| Ala | GCA(0.96) | GCC(0.92) | GCG(0.49) | GCT(1.63) |  |  |
| Arg | AGA(1.41) | AGG(0.79) | CGA(1.32) | CGC(0.53) | CGG(0.69) | CGT(1.26) |
| Asn | AAC(0.63) | AAT(1.37) |  |  |  |  |
| Asp | GAC(0.64) | GAT(1.36) |  |  |  |  |
| Cys | TGC(0.76) | TGT(1.24) |  |  |  |  |
| End | TAA(1.22) | TAG(1.17) | TGA(0.61) |  |  |  |
| Gln | CAA(1.49) | CAG(0.51) |  |  |  |  |
| Glu | GAA(1.34) | GAG(0.66) |  |  |  |  |
| Gly | GGA(1.42) | GGC(0.52) | GGG(0.69) | GGT(1.36) |  |  |
| His | CAC(0.43) | CAT(1.57) |  |  |  |  |
| Ile | ATA(0.8) | ATC(0.88) | ATT(1.32) |  |  |  |
| Leu | CTA(0.87) | CTC(0.63) | CTG(0.61) | CTT(1.21) | TTA(1.53) | TTG(1.15) |
| Lys | AAA(1.21) | AAG(0.79) |  |  |  |  |
| Met | ATG(1.0) |  |  |  |  |  |
| Phe | TTC(0.85) | TTT(1.15) |  |  |  |  |
| Pro | CCA(1.08) | CCC(0.77) | CCG(0.64) | CCT(1.52) |  |  |
| Ser | AGC(0.69) | AGT(1.08) | TCA(1.02) | TCC(0.97) | TCG(0.86) | TCT(1.39) |
| Thr | ACA(0.98) | ACC(1.01) | ACG(0.6) | ACT(1.42) |  |  |
| Trp | TGG(1.0) |  |  |  |  |  |
| Tyr | TAC(0.52) | TAT(1.48) |  |  |  |  |
| Val | GTA(1.2) | GTC(0.8) | GTG(0.84) | GTT(1.16) |  |  |
