# Supplementary material for: Mitochondrial genome of Isatis indigotica reveals repeat-mediated recombination and phylogenetic insights in Cruciferae
Source: Front Plant Sci. 2025 Oct 15;16:1655810. doi: 10.3389/fpls.2025.1655810 (PMC12568568; doi:10.3389/fpls.2025.1655810)
Supplement: Supplementary file 5 [file Table5.docx]

**Table S3 | Characterization of SSRs in the Mitogenome.**

| **ID** | **SSR nr.** | **SSR type** | **SSR** | **size** | **start** | **end** |
| --- | --- | --- | --- | --- | --- | --- |
| mtDNA | 1 | p3 | (ACA)4 | 12 | 1123 | 1134 |
| mtDNA | 2 | p2 | (AG)5 | 10 | 2607 | 2616 |
| mtDNA | 3 | p1 | (A)11 | 11 | 20255 | 20265 |
| mtDNA | 4 | c | (T)10...(AGAAC)3 | 586 | 21577 | 22162 |
| mtDNA | 5 | c | (ACAG)3...(TTCC)3 | 367 | 24654 | 25020 |
| mtDNA | 6 | c | (TTTC)3...(AATGGG)3 | 554 | 29548 | 30101 |
| mtDNA | 7 | p3 | (AAG)4 | 12 | 41242 | 41253 |
| mtDNA | 8 | p1 | (T)10 | 10 | 51472 | 51481 |
| mtDNA | 9 | p4 | (AGTG)3 | 12 | 52878 | 52889 |
| mtDNA | 10 | p4 | (TCAT)3 | 12 | 58064 | 58075 |
| mtDNA | 11 | p4 | (AGTC)3 | 12 | 64664 | 64675 |
| mtDNA | 12 | c | (T)10...(AAGA)3 | 691 | 68619 | 69309 |
| mtDNA | 13 | c | (CTAGT)3...(AATG)4 | 575 | 71319 | 71893 |
| mtDNA | 14 | c | (C)11...(GCCG)3 | 234 | 75311 | 75544 |
| mtDNA | 15 | p3 | (CTA)4 | 12 | 76982 | 76993 |
| mtDNA | 16 | p1 | (T)12 | 12 | 81860 | 81871 |
| mtDNA | 17 | p1 | (T)10 | 10 | 87870 | 87879 |
| mtDNA | 18 | c | (CT)5...(AG)5 | 422 | 93447 | 93868 |
| mtDNA | 19 | p3 | (AAC)4 | 12 | 97813 | 97824 |
| mtDNA | 20 | c | (AACA)3...(GGA)4 | 1432 | 101745 | 103176 |
| mtDNA | 21 | p4 | (AAAG)3 | 12 | 104612 | 104623 |
| mtDNA | 22 | p2 | (TC)7 | 14 | 109496 | 109509 |
| mtDNA | 23 | p4 | (TGAG)3 | 12 | 120648 | 120659 |
| mtDNA | 24 | p4 | (CATT)3 | 12 | 123223 | 123234 |
| mtDNA | 25 | p2 | (AT)6 | 12 | 127498 | 127509 |
| mtDNA | 26 | p2 | (AT)6 | 12 | 134226 | 134237 |
| mtDNA | 27 | p4 | (AAAC)3 | 12 | 146564 | 146575 |
| mtDNA | 28 | p1 | (T)11 | 11 | 159175 | 159185 |
| mtDNA | 29 | p4 | (TGAA)3 | 12 | 160813 | 160824 |
| mtDNA | 30 | p3 | (GGT)4 | 12 | 166077 | 166088 |
| mtDNA | 31 | p3 | (CTT)4 | 12 | 169291 | 169302 |
| mtDNA | 32 | p4 | (AGAA)3 | 12 | 180455 | 180466 |
| mtDNA | 33 | p2 | (TA)8 | 16 | 184909 | 184924 |
| mtDNA | 34 | c | (A)13...(A)14 | 1451 | 187733 | 189183 |
| mtDNA | 35 | p5 | (ATCAT)3 | 15 | 195473 | 195487 |
| mtDNA | 36 | c | (T)10...(TA)8 | 58 | 203952 | 204009 |
| mtDNA | 37 | p4 | (TGAG)3 | 12 | 208363 | 208374 |
| mtDNA | 38 | p4 | (CATT)3 | 12 | 210938 | 210949 |
| mtDNA | 39 | c | (TAAA)3...(TCTT)3 | 165 | 218698 | 218862 |
| mtDNA | 40 | c | (T)11...(A)13 | 519 | 220731 | 221249 |
| mtDNA | 41 | p5 | (AAGAG)3 | 15 | 223019 | 223033 |
| mtDNA | 42 | c | (GA)6...(TC)5 | 548 | 224483 | 225030 |
| mtDNA | 43 | p3 | (CTT)7 | 21 | 227943 | 227963 |
| mtDNA | 44 | p1 | (C)10 | 10 | 232937 | 232946 |
| mtDNA | 45 | p2 | (TC)5 | 10 | 243295 | 243304 |
| mtDNA | 46 | p2 | (AT)5 | 10 | 245062 | 245071 |
| mtDNA | 47 | p4 | (AAAC)3 | 12 | 250465 | 250476 |
| mtDNA | 48 | p4 | (AAAC)3 | 12 | 254147 | 254158 |
| mtDNA | 49 | p5 | (TTTTC)3 | 15 | 255489 | 255503 |
| mtDNA | 50 | p1 | (C)10 | 10 | 256858 | 256867 |
| mtDNA | 51 | c | (A)11...(T)10 | 377 | 258555 | 258931 |
