# Supplementary material for: Mitochondrial genome of Isatis indigotica reveals repeat-mediated recombination and phylogenetic insights in Cruciferae
Source: Front Plant Sci. 2025 Oct 15;16:1655810. doi: 10.3389/fpls.2025.1655810 (PMC12568568; doi:10.3389/fpls.2025.1655810)
Supplement: Supplementary file 6 [file Table6.docx]

**Table S4 | Identification of Tandem Repeats in the Mitochondrial Genome**

| ID | **Indices** | **Period Size** | **Copy Number** | **Consensus Size** | **Percent Matches** | **Percent Indels** | **Score** | **A** | **C** | **G** | **T** | **Entropy (0-2)** |
| --- | --- | --- | --- | --- | --- | --- | --- | --- | --- | --- | --- | --- |
| mtDNA | 3351—3423 | 35 | 2.1 | 35 | 100 | 0 | 146 | 45 | 17 | 21 | 15 | 1.85 |
| mtDNA | 4240—4406 | 41 | 3.9 | 41 | 84 | 12 | 187 | 31 | 11 | 32 | 23 | 1.91 |
| mtDNA | 4237—4406 | 22 | 7.9 | 22 | 85 | 10 | 226 | 31 | 11 | 32 | 23 | 1.91 |
| mtDNA | 4237—4406 | 63 | 2.6 | 64 | 94 | 4 | 288 | 31 | 11 | 32 | 23 | 1.91 |
| mtDNA | 31987—32025 | 19 | 2.1 | 19 | 95 | 0 | 69 | 33 | 30 | 15 | 20 | 1.94 |
| mtDNA | 47230—47265 | 18 | 2 | 18 | 100 | 0 | 72 | 44 | 16 | 0 | 38 | 1.48 |
| mtDNA | 50215—50252 | 19 | 2 | 19 | 100 | 0 | 76 | 10 | 26 | 10 | 52 | 1.68 |
| mtDNA | 58978—59051 | 27 | 2.7 | 28 | 95 | 2 | 132 | 14 | 16 | 24 | 44 | 1.85 |
| mtDNA | 92318—92351 | 17 | 2 | 17 | 100 | 0 | 68 | 35 | 29 | 0 | 35 | 1.58 |
| mtDNA | 95294—95320 | 12 | 2.2 | 12 | 100 | 0 | 54 | 11 | 29 | 37 | 22 | 1.89 |
| mtDNA | 96775—96859 | 36 | 2.4 | 36 | 100 | 0 | 170 | 22 | 21 | 18 | 37 | 1.94 |
| mtDNA | 109389—109456 | 34 | 2 | 34 | 88 | 0 | 100 | 26 | 29 | 26 | 17 | 1.98 |
| mtDNA | 112664—112720 | 24 | 2.4 | 24 | 94 | 2 | 98 | 42 | 14 | 14 | 29 | 1.84 |
| mtDNA | 115801—115838 | 20 | 1.9 | 20 | 100 | 0 | 76 | 44 | 21 | 7 | 26 | 1.79 |
| mtDNA | 115954—116040 | 36 | 2.4 | 36 | 94 | 1 | 149 | 28 | 26 | 16 | 28 | 1.97 |
| mtDNA | 118582—118644 | 27 | 2.3 | 27 | 100 | 0 | 126 | 33 | 12 | 31 | 22 | 1.91 |
| mtDNA | 130328—130371 | 10 | 4.7 | 10 | 81 | 13 | 58 | 9 | 20 | 59 | 11 | 1.59 |
| mtDNA | 130331—130368 | 18 | 2.1 | 18 | 95 | 0 | 67 | 7 | 21 | 60 | 10 | 1.54 |
| mtDNA | 133231—133269 | 17 | 2.3 | 17 | 95 | 0 | 69 | 41 | 10 | 15 | 33 | 1.81 |
| mtDNA | 140789—140821 | 14 | 2.2 | 16 | 89 | 10 | 52 | 15 | 30 | 6 | 48 | 1.69 |
| mtDNA | 141187—141214 | 14 | 2 | 14 | 100 | 0 | 56 | 14 | 21 | 21 | 42 | 1.88 |
| mtDNA | 155412—155460 | 22 | 2.2 | 22 | 96 | 0 | 89 | 36 | 20 | 20 | 22 | 1.95 |
| mtDNA | 173668—173764 | 45 | 2.2 | 45 | 98 | 0 | 185 | 19 | 25 | 17 | 37 | 1.94 |
| mtDNA | 206297—206359 | 27 | 2.3 | 27 | 100 | 0 | 126 | 33 | 12 | 31 | 22 | 1.91 |
| mtDNA | 211396—211429 | 15 | 2.3 | 15 | 100 | 0 | 68 | 14 | 35 | 44 | 5 | 1.7 |
| mtDNA | 216990—217046 | 28 | 2 | 28 | 100 | 0 | 114 | 12 | 35 | 3 | 49 | 1.58 |
| mtDNA | 239199—239247 | 23 | 2.1 | 23 | 100 | 0 | 98 | 16 | 6 | 24 | 53 | 1.66 |
| mtDNA | 239212—239250 | 11 | 3.5 | 11 | 79 | 6 | 51 | 15 | 5 | 25 | 53 | 1.62 |
| mtDNA | 244195—244219 | 12 | 2.1 | 12 | 100 | 0 | 50 | 28 | 24 | 16 | 32 | 1.96 |
| mtDNA | 253057—253098 | 18 | 2.4 | 18 | 84 | 11 | 52 | 28 | 33 | 14 | 23 | 1.94 |
