# Supplementary material for: Mitochondrial genome of Isatis indigotica reveals repeat-mediated recombination and phylogenetic insights in Cruciferae
Source: Front Plant Sci. 2025 Oct 15;16:1655810. doi: 10.3389/fpls.2025.1655810 (PMC12568568; doi:10.3389/fpls.2025.1655810)
Supplement: Supplementary file 7 [file Table7.docx]

**Table S5 |** **Identification of Large Repeats in the Mitogenome.**

| **ID** | **Repeat length** | **The start site of 1st part** | **The end site of 1st part** | **Type** | **The start site of 2nd part** | **The end site of 2^nd^ part** | **E-value** |
| --- | --- | --- | --- | --- | --- | --- | --- |
| Frepeat6329 | 6329 | 117231 | 123559 | F | 204946 | 211274 | 0.00E+00 |
| Frepeat738 | 738 | 161903 | 162640 | F | 260126 | 260863 | 0.00E+00 |
| Prepeat601 | 601 | 54512 | 55112 | P | 236355 | 236955 | 0.00E+00 |
| Prepeat583 | 583 | 102322 | 102904 | P | 190690 | 191272 | 0.00E+00 |
| Prepeat326 | 326 | 61184 | 61509 | P | 170557 | 170882 | 1.02E-186 |
| Frepeat146 | 146 | 109049 | 109194 | F | 149250 | 149395 | 2.41E-78 |
| Prepeat135 | 135 | 61151 | 61285 | P | 196546 | 196680 | 1.01E-71 |
| Frepeat105 | 105 | 102967 | 103071 | F | 125085 | 125189 | 1.16E-53 |
| Frepeat102 | 102 | 170781 | 170882 | F | 196546 | 196647 | 7.44E-52 |
| Frepeat96 | 96 | 144213 | 144308 | F | 242444 | 242539 | 3.05E-48 |
| Prepeat93 | 93 | 55381 | 55473 | P | 259865 | 259957 | 1.95E-46 |
| Frepeat89 | 89 | 199787 | 199875 | F | 212874 | 212962 | 5.00E-44 |
| Frepeat71 | 71 | 23758 | 23828 | F | 220462 | 220532 | 3.43E-33 |
| Frepeat71 | 71 | 102903 | 102973 | F | 124714 | 124784 | 3.43E-33 |
| Frepeat69 | 69 | 184177 | 184245 | F | 198890 | 198958 | 5.49E-32 |
| Prepeat68 | 68 | 89820 | 89887 | P | 155470 | 155537 | 2.20E-31 |
| Prepeat63 | 63 | 61394 | 61456 | P | 224958 | 225020 | 2.25E-28 |
| Frepeat63 | 63 | 170610 | 170672 | F | 224958 | 225020 | 2.25E-28 |
| Prepeat62 | 62 | 61341 | 61402 | P | 148198 | 148259 | 9.00E-28 |
| Frepeat62 | 62 | 148198 | 148259 | F | 170664 | 170725 | 9.00E-28 |
| Frepeat59 | 59 | 4236 | 4294 | F | 4299 | 4357 | 5.76E-26 |
| Prepeat57 | 57 | 61288 | 61344 | P | 225055 | 225111 | 9.22E-25 |
| Frepeat57 | 57 | 170722 | 170778 | F | 225055 | 225111 | 9.22E-25 |
| Frepeat56 | 56 | 237596 | 237651 | F | 241656 | 241711 | 3.69E-24 |
| Prepeat54 | 54 | 1721 | 1774 | P | 72413 | 72466 | 5.90E-23 |
| Frepeat52 | 52 | 109258 | 109309 | F | 149448 | 149499 | 9.44E-22 |
| Frepeat50 | 50 | 1883 | 1932 | F | 217522 | 217571 | 1.51E-20 |
| Frepeat49 | 49 | 96774 | 96822 | F | 96810 | 96858 | 6.04E-20 |
| Prepeat49 | 49 | 184445 | 184493 | P | 233504 | 233552 | 6.04E-20 |
| Frepeat47 | 47 | 173667 | 173713 | F | 173712 | 173758 | 9.66E-19 |
| Prepeat46 | 46 | 72622 | 72667 | P | 89548 | 89593 | 3.87E-18 |
| Frepeat45 | 45 | 13856 | 13900 | F | 62550 | 62594 | 1.55E-17 |
| Frepeat43 | 43 | 86485 | 86527 | F | 186952 | 186994 | 2.47E-16 |
| Prepeat43 | 43 | 196170 | 196212 | P | 199247 | 199289 | 2.47E-16 |
| Prepeat42 | 42 | 32075 | 32116 | P | 114546 | 114587 | 9.89E-16 |
| Prepeat42 | 42 | 34899 | 34940 | P | 83864 | 83905 | 9.89E-16 |
| Prepeat42 | 42 | 72571 | 72612 | P | 89597 | 89638 | 9.89E-16 |
| Frepeat42 | 42 | 109424 | 109465 | F | 111264 | 111305 | 9.89E-16 |
| Frepeat41 | 41 | 46317 | 46357 | F | 81892 | 81932 | 3.96E-15 |
| Frepeat41 | 41 | 72290 | 72330 | F | 186137 | 186177 | 3.96E-15 |
| Frepeat40 | 40 | 32077 | 32116 | F | 173316 | 173355 | 1.58E-14 |
| Prepeat40 | 40 | 114546 | 114585 | P | 173316 | 173355 | 1.58E-14 |
| Prepeat40 | 40 | 126882 | 126921 | P | 155467 | 155506 | 1.58E-14 |
| Frepeat39 | 39 | 25604 | 25642 | F | 155565 | 155603 | 6.33E-14 |
| Frepeat39 | 39 | 53900 | 53938 | F | 155566 | 155604 | 6.33E-14 |
| Prepeat39 | 39 | 61457 | 61495 | P | 224920 | 224958 | 6.33E-14 |
| Frepeat39 | 39 | 170571 | 170609 | F | 224920 | 224958 | 6.33E-14 |
| Frepeat38 | 38 | 3350 | 3387 | F | 3385 | 3422 | 2.53E-13 |
| Frepeat38 | 38 | 25605 | 25642 | F | 53900 | 53937 | 2.53E-13 |
| Frepeat38 | 38 | 34600 | 34637 | P | 182999 | 183036 | 2.53E-13 |
