# Supplementary material for: Mitochondrial genome of Isatis indigotica reveals repeat-mediated recombination and phylogenetic insights in Cruciferae
Source: Front Plant Sci. 2025 Oct 15;16:1655810. doi: 10.3389/fpls.2025.1655810 (PMC12568568; doi:10.3389/fpls.2025.1655810)
Supplement: Supplementary file 8 [file Table8.docx]

**Table S6 | Identification of Mitochondrial Plastid DNA Sequences (MTPTs) in the Mitogenome.**

| **ID** | **Query** | **Object** | **Identity (%)** | **Length (bp)** | **Mismatches** | **Gap Openings** | **Alignment Start (Plastome)** | **Alignment End (Plastome)** | **Alignment Start (Mitogenome)** | **Alignment End (Mitogenome)** | **E-value** | **Bit Score** | **MTPT annotation** |
| --- | --- | --- | --- | --- | --- | --- | --- | --- | --- | --- | --- | --- | --- |
| MTPT1 | cpDNA | mtDNA | 97.952 | 1367 | 21 | 2 | 54076 | 55435 | 191273 | 192639 | 0 | 2362 | rbcL |
| MTPT2 | cpDNA | mtDNA | 95.486 | 1019 | 26 | 3 | 67766 | 68764 | 46401 | 47419 | 0 | 1609 | psaB |
| MTPT3 | cpDNA | mtDNA | 99.813 | 536 | 1 | 0 | 134563 | 135098 | 161269 | 161804 | 0 | 985 | ycf1 |
| MTPT4 | cpDNA | mtDNA | 99.813 | 536 | 1 | 0 | 768 | 1303 | 161804 | 161269 | 0 | 985 | ycf1 |
| MTPT5 | cpDNA | mtDNA | 97.403 | 231 | 5 | 1 | 130214 | 130443 | 94583 | 94813 | 2.61E-108 | 392 | rrn23 |
| MTPT6 | cpDNA | mtDNA | 97.403 | 231 | 5 | 1 | 5423 | 5652 | 94813 | 94583 | 2.61E-108 | 392 | rrn23 |
| MTPT7 | cpDNA | mtDNA | 74.374 | 878 | 170 | 43 | 8253 | 9105 | 33565 | 34412 | 9.64E-88 | 324 | rrn16 |
| MTPT8 | cpDNA | mtDNA | 74.374 | 878 | 170 | 43 | 126761 | 127613 | 34412 | 33565 | 9.64E-88 | 324 | rrn16 |
| MTPT9 | cpDNA | mtDNA | 98.561 | 139 | 2 | 0 | 135583 | 135721 | 243847 | 243985 | 2.15E-64 | 246 | ycf1 |
| MTPT10 | cpDNA | mtDNA | 98.561 | 139 | 2 | 0 | 145 | 283 | 243985 | 243847 | 2.15E-64 | 246 | ycf1 |
| MTPT11 | cpDNA | mtDNA | 97.802 | 91 | 2 | 0 | 70147 | 70237 | 58896 | 58806 | 1.03E-37 | 158 | psaB |
| MTPT12 | cpDNA | mtDNA | 88.983 | 118 | 13 | 0 | 43806 | 43923 | 220952 | 221069 | 2.24E-34 | 147 | trnP-UGG |
| MTPT13 | cpDNA | mtDNA | 98.795 | 83 | 0 | 1 | 44114 | 44196 | 221284 | 221365 | 2.24E-34 | 147 | trnW-CCA |
| MTPT14 | cpDNA | mtDNA | 97.297 | 74 | 2 | 0 | 79691 | 79764 | 115797 | 115724 | 2.92E-28 | 126 | trnD-GUC |
| MTPT15 | cpDNA | mtDNA | 92.208 | 77 | 6 | 0 | 58008 | 58084 | 151573 | 151649 | 2.94E-23 | 110 | trnM-CAU |
| MTPT16 | cpDNA | mtDNA | 85.526 | 76 | 7 | 3 | 23921 | 23995 | 229258 | 229330 | 2.98E-13 | 76.8 | trnM-CAU |
| MTPT17 | cpDNA | mtDNA | 85.526 | 76 | 7 | 3 | 111871 | 111945 | 229330 | 229258 | 2.98E-13 | 76.8 | trnI-CAU |
