# Supplementary material for: Mitochondrial genome of Isatis indigotica reveals repeat-mediated recombination and phylogenetic insights in Cruciferae
Source: Front Plant Sci. 2025 Oct 15;16:1655810. doi: 10.3389/fpls.2025.1655810 (PMC12568568; doi:10.3389/fpls.2025.1655810)
Supplement: Supplementary file 9 [file Table9.docx]

**Table S7 | Comprehensive Summary of C-to-U RNA Editing Events and Their Associated Amino Acid Changes in Mitochondrial Genes.**

| Gene | Base | Aa | Triplet pos. | Bases | Codon | Aa change | LABEL |
| --- | --- | --- | --- | --- | --- | --- | --- |
| nad7 | 244 | 82 | 1 | C→U | CAU→UAU | H→Y | 24482 |
| nad7 | 251 | 84 | 2 | C→U | UCA→UUA | S→L | 25184 |
| nad7 | 316 | 106 | 1 | C→U | CGU→UGU | R→C | 316106 |
| nad7 | 335 | 112 | 2 | C→U | UCA→UUA | S→L | 335112 |
| nad7 | 344 | 115 | 2 | C→U | UCA→UUA | S→L | 344115 |
| nad7 | 578 | 193 | 2 | C→U | UCA→UUA | S→L | 578193 |
| nad7 | 1057 | 353 | 1 | C→U | CGU→UGU | R→C | 1057353 |
| nad7 | 1079 | 360 | 2 | C→U | UCU→UUU | S→F | 1079360 |
| nad7 | 1088 | 363 | 2 | C→U | UCA→UUA | S→L | 1088363 |
| nad7 | 1103 | 368 | 2 | C→U | UCU→UUU | S→F | 1103368 |
| nad7 | 1124 | 375 | 2 | C→U | CCA→CUA | P→L | 1124375 |
| nad7 | 698 | 233 | 2 | C→U | UCG→UUG | S→L | 698233 |
| nad7 | 724 | 242 | 1 | C→U | CAU→UAU | H→Y | 724242 |
| nad7 | 734 | 245 | 2 | C→U | UCG→UUG | S→L | 734245 |
| nad7 | 769 | 257 | 1 | C→U | CGC→UGC | R→C | 769257 |
| nad7 | 38 | 13 | 2 | C→U | UCG→UUG | S→L | 3813 |
| nad7 | 77 | 26 | 2 | C→U | UCA→UUA | S→L | 7726 |
| nad7 | 137 | 46 | 2 | C→U | UCA→UUA | S→L | 13746 |
| nad7 | 200 | 67 | 2 | C→U | UCU→UUU | S→F | 20067 |
| atp8 | 389 | 130 | 2 | C→U | UCA→UUA | S→L | 389130 |
| nad5 | 242 | 81 | 2 | C→U | CCG→CUG | P→L | 24281 |
| nad5 | 272 | 91 | 2 | C→U | UCC→UUC | S→F | 27291 |
| nad5 | 358 | 120 | 1 | C→U | CUU→UUU | L→F | 358120 |
| nad5 | 374 | 125 | 2 | C→U | CCA→CUA | P→L | 374125 |
| nad5 | 398 | 133 | 2 | C→U | UCU→UUU | S→F | 398133 |
| nad5 | 494 | 165 | 2 | C→U | ACA→AUA | T→I | 494165 |
| nad5 | 539 | 180 | 2 | C→U | CCU→CUU | P→L | 539180 |
| nad5 | 548 | 183 | 2 | C→U | UCG→UUG | S→L | 548183 |
| nad5 | 553 | 185 | 1 | C→U | CGU→UGU | R→C | 553185 |
| nad5 | 598 | 200 | 1 | C→U | CGU→UGU | R→C | 598200 |
| nad5 | 608 | 203 | 2 | C→U | GCC→GUC | A→V | 608203 |
| nad5 | 629 | 210 | 2 | C→U | UCU→UUU | S→F | 629210 |
| nad5 | 676 | 226 | 1 | C→U | CUU→UUU | L→F | 676226 |
| nad5 | 713 | 238 | 2 | C→U | UCG→UUG | S→L | 713238 |
| nad5 | 725 | 242 | 2 | C→U | UCA→UUA | S→L | 725242 |
| nad5 | 764 | 255 | 2 | C→U | UCG→UUG | S→L | 764255 |
| nad5 | 835 | 279 | 1 | C→U | CCA→UCA | P→S | 835279 |
| nad5 | 863 | 288 | 2 | C→U | UCU→UUU | S→F | 863288 |
| nad5 | 875 | 292 | 2 | C→U | ACG→AUG | T→M | 875292 |
| nad5 | 1400 | 467 | 2 | C→U | UCA→UUA | S→L | 1400467 |
| nad5 | 1490 | 497 | 2 | C→U | CCC→CUC | P→L | 1490497 |
| nad5 | 1550 | 517 | 2 | C→U | ACC→AUC | T→I | 1550517 |
| nad5 | 1580 | 527 | 2 | C→U | UCA→UUA | S→L | 1580527 |
| nad5 | 1610 | 537 | 2 | C→U | CCC→CUC | P→L | 1610537 |
| nad5 | 155 | 52 | 2 | C→U | CCG→CUG | P→L | 15552 |
| nad5 | 1895 | 632 | 2 | C→U | UCA→UUA | S→L | 1895632 |
| nad5 | 1916 | 639 | 2 | C→U | UCU→UUU | S→F | 1916639 |
| nad5 | 1918 | 640 | 1 | C→U | CGU→UGU | R→C | 1918640 |
| nad5 | 1958 | 653 | 2 | C→U | UCG→UUG | S→L | 1958653 |
| nad9 | 14 | 5 | 2 | C→U | UCC→UUC | S→F | 145 |
| nad9 | 92 | 31 | 2 | C→U | UCU→UUU | S→F | 9231 |
| nad9 | 167 | 56 | 2 | C→U | UCG→UUG | S→L | 16756 |
| nad9 | 298 | 100 | 1 | C→U | CCG→UCG | P→S | 298100 |
| nad9 | 328 | 110 | 1 | C→U | CGG→UGG | R→W | 328110 |
| nad9 | 398 | 133 | 2 | C→U | UCA→UUA | S→L | 398133 |
| nad9 | 439 | 147 | 1 | C→U | CUU→UUU | L→F | 439147 |
| ccmFN | 89 | 30 | 2 | C→U | UCG→UUG | S→L | 8930 |
| ccmFN | 200 | 67 | 2 | C→U | CCA→CUA | P→L | 20067 |
| ccmFN | 232 | 78 | 1 | C→U | CGG→UGG | R→W | 23278 |
| ccmFN | 250 | 84 | 1 | C→U | CGG→UGG | R→W | 25084 |
| ccmFN | 283 | 95 | 1 | C→U | CGG→UGG | R→W | 28395 |
| ccmFN | 301 | 101 | 1 | C→U | CGU→UGU | R→C | 301101 |
| ccmFN | 344 | 115 | 2 | C→U | UCG→UUG | S→L | 344115 |
| ccmFN | 368 | 123 | 2 | C→U | CCA→CUA | P→L | 368123 |
| ccmFN | 380 | 127 | 2 | C→U | UCA→UUA | S→L | 380127 |
| ccmFN | 415 | 139 | 1 | C→U | CCC→UCC | P→S | 415139 |
| atp6 | 103 | 35 | 1 | C→U | CCA→UCA | P→S | 10335 |
| atp4 | 89 | 30 | 2 | C→U | UCA→UUA | S→L | 8930 |
| atp4 | 215 | 72 | 2 | C→U | UCG→UUG | S→L | 21572 |
| atp4 | 248 | 83 | 2 | C→U | CCU→CUU | P→L | 24883 |
| atp4 | 251 | 84 | 2 | C→U | CCG→CUG | P→L | 25184 |
| atp4 | 395 | 132 | 2 | C→U | UCA→UUA | S→L | 395132 |
| atp4 | 416 | 139 | 2 | C→U | ACU→AUU | T→I | 416139 |
| nad4L | 8 | 3 | 2 | C→U | CCU→CUU | P→L | 83 |
| nad4L | 41 | 14 | 2 | C→U | UCU→UUU | S→F | 4114 |
| nad4L | 55 | 19 | 1 | C→U | CGG→UGG | R→W | 5519 |
| nad4L | 86 | 29 | 2 | C→U | CCU→CUU | P→L | 8629 |
| nad4L | 95 | 32 | 2 | C→U | UCA→UUA | S→L | 9532 |
| nad4L | 100 | 34 | 1 | C→U | CCA→UCA | P→S | 10034 |
| nad4L | 110 | 37 | 2 | C→U | UCA→UUA | S→L | 11037 |
| nad4L | 131 | 44 | 2 | C→U | UCG→UUG | S→L | 13144 |
| nad4L | 158 | 53 | 2 | C→U | UCG→UUG | S→L | 15853 |
| rps7 | 332 | 111 | 2 | C→U | UCA→UUA | S→L | 332111 |
| nad1 | 2 | 1 | 2 | C→U | ACG→AUG | T→M | 21 |
| nad1 | 167 | 56 | 2 | C→U | UCG→UUG | S→L | 16756 |
| nad1 | 265 | 89 | 1 | C→U | CGG→UGG | R→W | 26589 |
| nad1 | 308 | 103 | 2 | C→U | CCG→CUG | P→L | 308103 |
| nad1 | 376 | 126 | 1 | C→U | CGG→UGG | R→W | 376126 |
| nad1 | 725 | 242 | 2 | C→U | CCA→CUA | P→L | 725242 |
| nad1 | 743 | 248 | 2 | C→U | CCA→CUA | P→L | 743248 |
| nad1 | 755 | 252 | 2 | C→U | CCG→CUG | P→L | 755252 |
| nad1 | 823 | 275 | 1 | C→U | CUC→UUC | L→F | 823275 |
| nad1 | 898 | 300 | 1 | C→U | CGG→UGG | R→W | 898300 |
| nad1 | 928 | 310 | 1 | C→U | CGG→UGG | R→W | 928310 |
| nad1 | 937 | 313 | 1 | C→U | CCU→UCU | P→S | 937313 |
| nad1 | 490 | 164 | 1 | C→U | CCC→UCC | P→S | 490164 |
| nad1 | 493 | 165 | 1 | C→U | CGU→UGU | R→C | 493165 |
| nad1 | 500 | 167 | 2 | C→U | UCG→UUG | S→L | 500167 |
| nad1 | 536 | 179 | 2 | C→U | UCU→UUU | S→F | 536179 |
| nad1 | 571 | 191 | 1 | C→U | CUU→UUU | L→F | 571191 |
| nad1 | 580 | 194 | 1 | C→U | CGU→UGU | R→C | 580194 |
| nad1 | 635 | 212 | 2 | C→U | UCA→UUA | S→L | 635212 |
| nad1 | 761 | 254 | 2 | C→U | ACU→AUU | T→I | 761254 |
| nad1 | 935 | 312 | 2 | C→U | GCC→GUC | A→V | 935312 |
| matR | 386 | 129 | 2 | C→U | CCA→CUA | P→L | 386129 |
| matR | 473 | 158 | 2 | C→U | UCG→UUG | S→L | 473158 |
| matR | 1745 | 582 | 2 | C→U | UCC→UUC | S→F | 1745582 |
| matR | 1766 | 589 | 2 | C→U | CCU→CUU | P→L | 1766589 |
| matR | 1786 | 596 | 1 | C→U | CGC→UGC | R→C | 1786596 |
| matR | 1822 | 608 | 1 | C→U | CAC→UAC | H→Y | 1822608 |
| matR | 1892 | 631 | 2 | C→U | CCC→CUC | P→L | 1892631 |
| matR | 1910 | 637 | 2 | C→U | UCA→UUA | S→L | 1910637 |
| cox2 | 71 | 24 | 2 | C→U | UCU→UUU | S→F | 7124 |
| cox2 | 253 | 85 | 1 | C→U | CGG→UGG | R→W | 25385 |
| cox2 | 278 | 93 | 2 | C→U | UCG→UUG | S→L | 27893 |
| cox2 | 379 | 127 | 1 | C→U | CGG→UGG | R→W | 379127 |
| cox2 | 476 | 159 | 2 | C→U | UCA→UUA | S→L | 476159 |
| cox2 | 557 | 186 | 2 | C→U | CCU→CUU | P→L | 557186 |
| cox2 | 581 | 194 | 2 | C→U | UCA→UUA | S→L | 581194 |
| ccmFC | 50 | 17 | 2 | C→U | CCU→CUU | P→L | 5017 |
| ccmFC | 103 | 35 | 1 | C→U | CCC→UCC | P→S | 10335 |
| ccmFC | 119 | 40 | 2 | C→U | UCU→UUU | S→F | 11940 |
| ccmFC | 146 | 49 | 2 | C→U | CCU→CUU | P→L | 14649 |
| ccmFC | 155 | 52 | 2 | C→U | UCA→UUA | S→L | 15552 |
| ccmFC | 160 | 54 | 1 | C→U | CCU→UCU | P→S | 16054 |
| ccmFC | 334 | 112 | 1 | C→U | CGU→UGU | R→C | 334112 |
| ccmFC | 406 | 136 | 1 | C→U | CGU→UGU | R→C | 406136 |
| ccmFC | 415 | 139 | 1 | C→U | CUC→UUC | L→F | 415139 |
| ccmFC | 776 | 259 | 2 | C→U | UCA→UUA | S→L | 776259 |
| ccmFC | 890 | 297 | 2 | C→U | UCU→UUU | S→F | 890297 |
| ccmFC | 1172 | 391 | 2 | C→U | UCG→UUG | S→L | 1172391 |
| ccmFC | 1246 | 416 | 1 | C→U | CGG→UGG | R→W | 1246416 |
| ccmFC | 1280 | 427 | 2 | C→U | UCG→UUG | S→L | 1280427 |
| ccmFC | 1327 | 443 | 1 | C→U | CGA→UGA | R→* | 1327443 |
| cox3 | 112 | 38 | 1 | C→U | CCA→UCA | P→S | 11238 |
| cox3 | 245 | 82 | 2 | C→U | CCU→CUU | P→L | 24582 |
| cox3 | 257 | 86 | 2 | C→U | UCU→UUU | S→F | 25786 |
| cox3 | 311 | 104 | 2 | C→U | UCU→UUU | S→F | 311104 |
| cox3 | 314 | 105 | 2 | C→U | UCU→UUU | S→F | 314105 |
| cox3 | 413 | 138 | 2 | C→U | CCU→CUU | P→L | 413138 |
| cox3 | 422 | 141 | 2 | C→U | CCU→CUU | P→L | 422141 |
| ccmFN | 38 | 13 | 2 | C→U | CCG→CUG | P→L | 3813 |
| ccmFN | 98 | 33 | 2 | C→U | CCU→CUU | P→L | 9833 |
| ccmFN | 137 | 46 | 2 | C→U | UCG→UUG | S→L | 13746 |
| ccmFN | 151 | 51 | 1 | C→U | CCU→UCU | P→S | 15151 |
| ccmFN | 256 | 86 | 1 | C→U | CGG→UGG | R→W | 25686 |
| ccmFN | 263 | 88 | 2 | C→U | CCA→CUA | P→L | 26388 |
| ccmFN | 283 | 95 | 1 | C→U | CUU→UUU | L→F | 28395 |
| ccmFN | 776 | 259 | 2 | C→U | UCA→UUA | S→L | 776259 |
| ccmFN | 788 | 263 | 2 | C→U | CCA→CUA | P→L | 788263 |
| ccmFN | 803 | 268 | 2 | C→U | UCA→UUA | S→L | 803268 |
| ccmFN | 952 | 318 | 1 | C→U | CGC→UGC | R→C | 952318 |
| ccmFN | 976 | 326 | 1 | C→U | CGU→UGU | R→C | 976326 |
| atp9 | 20 | 7 | 2 | C→U | UCA→UUA | S→L | 207 |
| atp9 | 50 | 17 | 2 | C→U | UCA→UUA | S→L | 5017 |
| atp9 | 134 | 45 | 2 | C→U | UCA→UUA | S→L | 13445 |
| atp9 | 191 | 64 | 2 | C→U | CCA→CUA | P→L | 19164 |
| ccmFC | 44 | 15 | 2 | C→U | GCC→GUC | A→V | 4415 |
| ccmFC | 86 | 29 | 2 | C→U | GCU→GUU | A→V | 8629 |
| ccmFC | 203 | 68 | 2 | C→U | GCC→GUC | A→V | 20368 |
| ccmFC | 209 | 70 | 2 | C→U | UCC→UUC | S→F | 20970 |
| nad3 | 5 | 2 | 2 | C→U | UCA→UUA | S→L | 52 |
| nad3 | 23 | 8 | 2 | C→U | UCU→UUU | S→F | 238 |
| nad3 | 80 | 27 | 2 | C→U | CCA→CUA | P→L | 8027 |
| nad3 | 146 | 49 | 2 | C→U | UCC→UUC | S→F | 14649 |
| nad3 | 247 | 83 | 1 | C→U | CCU→UCU | P→S | 24783 |
| nad3 | 251 | 84 | 2 | C→U | CCC→CUC | P→L | 25184 |
| nad3 | 344 | 115 | 2 | C→U | UCG→UUG | S→L | 344115 |
| nad3 | 349 | 117 | 1 | C→U | CGG→UGG | R→W | 349117 |
| ccmB | 146 | 49 | 2 | C→U | CCA→CUA | P→L | 14649 |
| ccmB | 16 | 6 | 1 | C→U | CUU→UUU | L→F | 166 |
| ccmB | 28 | 10 | 1 | C→U | CAU→UAU | H→Y | 2810 |
| ccmB | 43 | 15 | 1 | C→U | CCC→UCC | P→S | 4315 |
| ccmB | 71 | 24 | 2 | C→U | CCA→CUA | P→L | 7124 |
| ccmB | 80 | 27 | 2 | C→U | UCG→UUG | S→L | 8027 |
| ccmB | 128 | 43 | 2 | C→U | UCA→UUA | S→L | 12843 |
| ccmB | 137 | 46 | 2 | C→U | UCC→UUC | S→F | 13746 |
| ccmB | 149 | 50 | 2 | C→U | CCG→CUG | P→L | 14950 |
| ccmB | 154 | 52 | 1 | C→U | CGG→UGG | R→W | 15452 |
| ccmB | 160 | 54 | 1 | C→U | CCU→UCU | P→S | 16054 |
| ccmB | 164 | 55 | 2 | C→U | CCG→CUG | P→L | 16455 |
| ccmB | 172 | 58 | 1 | C→U | CCU→UCU | P→S | 17258 |
| ccmB | 179 | 60 | 2 | C→U | CCU→CUU | P→L | 17960 |
| ccmB | 181 | 61 | 1 | C→U | CCU→UCU | P→S | 18161 |
| ccmB | 286 | 96 | 1 | C→U | CGG→UGG | R→W | 28696 |
| ccmB | 304 | 102 | 1 | C→U | CGU→UGU | R→C | 304102 |
| ccmB | 338 | 113 | 2 | C→U | UCG→UUG | S→L | 338113 |
| ccmB | 367 | 123 | 1 | C→U | CGG→UGG | R→W | 367123 |
| ccmB | 380 | 127 | 2 | C→U | CCA→CUA | P→L | 380127 |
| ccmB | 424 | 142 | 1 | C→U | CGU→UGU | R→C | 424142 |
| ccmB | 428 | 143 | 2 | C→U | UCG→UUG | S→L | 428143 |
| ccmB | 467 | 156 | 2 | C→U | UCG→UUG | S→L | 467156 |
| ccmB | 476 | 159 | 2 | C→U | CCA→CUA | P→L | 476159 |
| ccmB | 485 | 162 | 2 | C→U | UCA→UUA | S→L | 485162 |
| ccmB | 512 | 171 | 2 | C→U | UCU→UUU | S→F | 512171 |
| ccmB | 514 | 172 | 1 | C→U | CGU→UGU | R→C | 514172 |
| ccmB | 551 | 184 | 2 | C→U | UCA→UUA | S→L | 551184 |
| ccmB | 554 | 185 | 2 | C→U | UCG→UUG | S→L | 554185 |
| ccmB | 566 | 189 | 2 | C→U | UCC→UUC | S→F | 566189 |
| ccmB | 569 | 190 | 2 | C→U | UCU→UUU | S→F | 569190 |
| ccmB | 596 | 199 | 2 | C→U | UCG→UUG | S→L | 596199 |
| rpl2 | 212 | 71 | 2 | C→U | CCA→CUA | P→L | 21271 |
| tatC | 25 | 9 | 1 | C→U | CAU→UAU | H→Y | 259 |
| tatC | 35 | 12 | 2 | C→U | UCG→UUG | S→L | 3512 |
| tatC | 73 | 25 | 1 | C→U | CGG→UGG | R→W | 7325 |
| tatC | 121 | 41 | 1 | C→U | CCG→UCG | P→S | 12141 |
| tatC | 137 | 46 | 2 | C→U | UCU→UUU | S→F | 13746 |
| tatC | 140 | 47 | 2 | C→U | CCA→CUA | P→L | 14047 |
| tatC | 149 | 50 | 2 | C→U | UCA→UUA | S→L | 14950 |
| tatC | 337 | 113 | 1 | C→U | CUC→UUC | L→F | 337113 |
| tatC | 340 | 114 | 1 | C→U | CAU→UAU | H→Y | 340114 |
| tatC | 353 | 118 | 2 | C→U | UCU→UUU | S→F | 353118 |
| tatC | 385 | 129 | 1 | C→U | CCC→UCC | P→S | 385129 |
| tatC | 388 | 130 | 1 | C→U | CGG→UGG | R→W | 388130 |
| tatC | 416 | 139 | 2 | C→U | CCA→CUA | P→L | 416139 |
| tatC | 481 | 161 | 1 | C→U | CAU→UAU | H→Y | 481161 |
| tatC | 506 | 169 | 2 | C→U | UCG→UUG | S→L | 506169 |
| tatC | 514 | 172 | 1 | C→U | CCA→UCA | P→S | 514172 |
| tatC | 550 | 184 | 1 | C→U | CGU→UGU | R→C | 550184 |
| tatC | 557 | 186 | 2 | C→U | CCA→CUA | P→L | 557186 |
| tatC | 563 | 188 | 2 | C→U | CCA→CUA | P→L | 563188 |
| tatC | 619 | 207 | 1 | C→U | CCG→UCG | P→S | 619207 |
| tatC | 625 | 209 | 1 | C→U | CUC→UUC | L→F | 625209 |
| tatC | 641 | 214 | 2 | C→U | UCC→UUC | S→F | 641214 |
| tatC | 676 | 226 | 1 | C→U | CGU→UGU | R→C | 676226 |
| tatC | 722 | 241 | 2 | C→U | UCG→UUG | S→L | 722241 |
| nad4 | 608 | 203 | 2 | C→U | UCA→UUA | S→L | 608203 |
| nad4 | 659 | 220 | 2 | C→U | UCU→UUU | S→F | 659220 |
| nad4 | 668 | 223 | 2 | C→U | UCU→UUU | S→F | 668223 |
| nad4 | 767 | 256 | 2 | C→U | CCU→CUU | P→L | 767256 |
| nad4 | 784 | 262 | 1 | C→U | CAC→UAC | H→Y | 784262 |
| nad4 | 836 | 279 | 2 | C→U | UCU→UUU | S→F | 836279 |
| nad4 | 896 | 299 | 2 | C→U | UCA→UUA | S→L | 896299 |
| nad4 | 29 | 10 | 2 | C→U | UCU→UUU | S→F | 2910 |
| nad4 | 74 | 25 | 2 | C→U | ACU→AUU | T→I | 7425 |
| nad4 | 77 | 26 | 2 | C→U | CCU→CUU | P→L | 7726 |
| nad4 | 107 | 36 | 2 | C→U | CCG→CUG | P→L | 10736 |
| nad4 | 158 | 53 | 2 | C→U | CCU→CUU | P→L | 15853 |
| nad4 | 164 | 55 | 2 | C→U | CCU→CUU | P→L | 16455 |
| nad4 | 166 | 56 | 1 | C→U | CGG→UGG | R→W | 16656 |
| nad4 | 197 | 66 | 2 | C→U | UCU→UUU | S→F | 19766 |
| nad4 | 317 | 106 | 2 | C→U | UCA→UUA | S→L | 317106 |
| nad4 | 362 | 121 | 2 | C→U | ACA→AUA | T→I | 362121 |
| nad4 | 376 | 126 | 1 | C→U | CGU→UGU | R→C | 376126 |
| nad4 | 403 | 135 | 1 | C→U | CGC→UGC | R→C | 403135 |
| nad4 | 1010 | 337 | 2 | C→U | CCG→CUG | P→L | 1010337 |
| nad4 | 1033 | 345 | 1 | C→U | CCU→UCU | P→S | 1033345 |
| nad4 | 1129 | 377 | 1 | C→U | CUC→UUC | L→F | 1129377 |
| nad4 | 1148 | 383 | 2 | C→U | UCU→UUU | S→F | 1148383 |
| nad4 | 1172 | 391 | 2 | C→U | UCA→UUA | S→L | 1172391 |
| nad4 | 1205 | 402 | 2 | C→U | CCC→CUC | P→L | 1205402 |
| nad4 | 1355 | 452 | 2 | C→U | UCA→UUA | S→L | 1355452 |
| nad4 | 1373 | 458 | 2 | C→U | UCC→UUC | S→F | 1373458 |
| nad4 | 1307 | 436 | 2 | C→U | GCA→GUA | A→V | 1307436 |
| nad4 | 1405 | 469 | 1 | C→U | CGG→UGG | R→W | 1405469 |
| nad4 | 1417 | 473 | 1 | C→U | CAC→UAC | H→Y | 1417473 |
| nad4 | 1433 | 478 | 2 | C→U | CCG→CUG | P→L | 1433478 |
| nad6 | 26 | 9 | 2 | C→U | CCU→CUU | P→L | 269 |
| nad6 | 53 | 18 | 2 | C→U | GCA→GUA | A→V | 5318 |
| nad6 | 95 | 32 | 2 | C→U | CCA→CUA | P→L | 9532 |
| nad6 | 103 | 35 | 1 | C→U | CGC→UGC | R→C | 10335 |
| nad6 | 161 | 54 | 2 | C→U | CCA→CUA | P→L | 16154 |
| nad6 | 169 | 57 | 1 | C→U | CAU→UAU | H→Y | 16957 |
| nad6 | 191 | 64 | 2 | C→U | UCA→UUA | S→L | 19164 |
| nad6 | 446 | 149 | 2 | C→U | UCU→UUU | S→F | 446149 |
| nad6 | 463 | 155 | 1 | C→U | CCU→UCU | P→S | 463155 |
| nad2 | 788 | 263 | 2 | C→U | UCU→UUU | S→F | 788263 |
| nad2 | 809 | 270 | 2 | C→U | UCU→UUU | S→F | 809270 |
| nad2 | 920 | 307 | 2 | C→U | CCU→CUU | P→L | 920307 |
| nad2 | 928 | 310 | 1 | C→U | CAU→UAU | H→Y | 928310 |
| nad2 | 958 | 320 | 1 | C→U | CGU→UGU | R→C | 958320 |
| nad2 | 962 | 321 | 2 | C→U | ACU→AUU | T→I | 962321 |
| nad2 | 1058 | 353 | 2 | C→U | UCA→UUA | S→L | 1058353 |
| nad2 | 1127 | 376 | 2 | C→U | UCG→UUG | S→L | 1127376 |
| nad2 | 1247 | 416 | 2 | C→U | CCA→CUA | P→L | 1247416 |
| nad2 | 1276 | 426 | 1 | C→U | CGU→UGU | R→C | 1276426 |
| nad2 | 308 | 103 | 2 | C→U | UCU→UUU | S→F | 308103 |
| nad2 | 311 | 104 | 2 | C→U | UCC→UUC | S→F | 311104 |
| nad2 | 356 | 119 | 2 | C→U | CCA→CUA | P→L | 356119 |
| nad2 | 361 | 121 | 1 | C→U | CCU→UCU | P→S | 361121 |
| nad2 | 367 | 123 | 1 | C→U | CGC→UGC | R→C | 367123 |
| nad2 | 394 | 132 | 1 | C→U | CAU→UAU | H→Y | 394132 |
| nad2 | 428 | 143 | 2 | C→U | CCU→CUU | P→L | 428143 |
| nad2 | 497 | 166 | 2 | C→U | UCG→UUG | S→L | 497166 |
| nad2 | 1298 | 433 | 2 | C→U | GCG→GUG | A→V | 1298433 |
| nad2 | 1400 | 467 | 2 | C→U | UCA→UUA | S→L | 1400467 |
| nad2 | 1403 | 468 | 2 | C→U | UCC→UUC | S→F | 1403468 |
| nad2 | 1457 | 486 | 2 | C→U | UCA→UUA | S→L | 1457486 |
| nad2 | 662 | 221 | 2 | C→U | UCU→UUU | S→F | 662221 |
| nad2 | 26 | 9 | 2 | C→U | UCC→UUC | S→F | 269 |
| nad2 | 56 | 19 | 2 | C→U | UCC→UUC | S→F | 5619 |
| rps3 | 1528 | 510 | 1 | C→U | CGU→UGU | R→C | 1528510 |
| rps3 | 1561 | 521 | 1 | C→U | CCU→UCU | P→S | 1561521 |
| rps3 | 1592 | 531 | 2 | C→U | UCA→UUA | S→L | 1592531 |
| rps3 | 64 | 22 | 1 | C→U | CGG→UGG | R→W | 6422 |
| rpl16 | 104 | 35 | 2 | C→U | ACU→AUU | T→I | 10435 |
| rpl16 | 335 | 112 | 2 | C→U | CCA→CUA | P→L | 335112 |
| rpl16 | 401 | 134 | 2 | C→U | CCA→CUA | P→L | 401134 |
| rpl16 | 407 | 136 | 2 | C→U | UCG→UUG | S→L | 407136 |
| rpl5 | 35 | 12 | 2 | C→U | UCA→UUA | S→L | 3512 |
| rpl5 | 47 | 16 | 2 | C→U | CCG→CUG | P→L | 4716 |
| rpl5 | 59 | 20 | 2 | C→U | CCG→CUG | P→L | 5920 |
| rpl5 | 64 | 22 | 1 | C→U | CAC→UAC | H→Y | 6422 |
| rpl5 | 92 | 31 | 2 | C→U | UCG→UUG | S→L | 9231 |
| rpl5 | 169 | 57 | 1 | C→U | CGC→UGC | R→C | 16957 |
| rpl5 | 317 | 106 | 2 | C→U | UCG→UUG | S→L | 317106 |
| rpl5 | 329 | 110 | 2 | C→U | UCG→UUG | S→L | 329110 |
| rpl5 | 512 | 171 | 2 | C→U | CCA→CUA | P→L | 512171 |
| rpl5 | 515 | 172 | 2 | C→U | CCG→CUG | P→L | 515172 |
| cob | 286 | 96 | 1 | C→U | CUU→UUU | L→F | 28696 |
| cob | 325 | 109 | 1 | C→U | CAU→UAU | H→Y | 325109 |
| cob | 568 | 190 | 1 | C→U | CAU→UAU | H→Y | 568190 |
| cob | 853 | 285 | 1 | C→U | CAU→UAU | H→Y | 853285 |
| cob | 908 | 303 | 2 | C→U | CCA→CUA | P→L | 908303 |
| cob | 982 | 328 | 1 | C→U | CAC→UAC | H→Y | 982328 |
| cob | 1084 | 362 | 1 | C→U | CCU→UCU | P→S | 1084362 |
| cox2 | 721 | 241 | 1 | C→U | CCU→UCU | P→S | 721241 |
| cox2 | 742 | 248 | 1 | C→U | CGG→UGG | R→W | 742248 |
| ccmC | 179 | 60 | 2 | C→U | GCG→GUG | A→V | 17960 |
| ccmC | 184 | 62 | 1 | C→U | CGG→UGG | R→W | 18462 |
| ccmC | 331 | 111 | 1 | C→U | CGG→UGG | R→W | 331111 |
| ccmC | 395 | 132 | 2 | C→U | UCG→UUG | S→L | 395132 |
| ccmC | 400 | 134 | 1 | C→U | CUU→UUU | L→F | 400134 |
| ccmC | 421 | 141 | 1 | C→U | CGU→UGU | R→C | 421141 |
| ccmC | 436 | 146 | 1 | C→U | CCU→UCU | P→S | 436146 |
| ccmC | 446 | 149 | 2 | C→U | CCG→CUG | P→L | 446149 |
| ccmC | 458 | 153 | 2 | C→U | UCA→UUA | S→L | 458153 |
| ccmC | 463 | 155 | 1 | C→U | CGU→UGU | R→C | 463155 |
| ccmC | 467 | 156 | 2 | C→U | GCU→GUU | A→V | 467156 |
| ccmC | 473 | 158 | 2 | C→U | CCG→CUG | P→L | 473158 |
| ccmC | 497 | 166 | 2 | C→U | UCU→UUU | S→F | 497166 |
| ccmC | 521 | 174 | 2 | C→U | UCG→UUG | S→L | 521174 |
| ccmC | 548 | 183 | 2 | C→U | UCU→UUU | S→F | 548183 |
| ccmC | 568 | 190 | 1 | C→U | CCU→UCU | P→S | 568190 |
| ccmC | 575 | 192 | 2 | C→U | CCC→CUC | P→L | 575192 |
| ccmC | 608 | 203 | 2 | C→U | CCC→CUC | P→L | 608203 |
| ccmC | 614 | 205 | 2 | C→U | UCA→UUA | S→L | 614205 |
| ccmC | 619 | 207 | 1 | C→U | CGU→UGU | R→C | 619207 |
| ccmC | 650 | 217 | 2 | C→U | CCU→CUU | P→L | 650217 |
| rps4 | 25 | 9 | 1 | C→U | CAA→UAA | Q→* | 259 |
| rps4 | 77 | 26 | 2 | C→U | UCA→UUA | S→L | 7726 |
| rps4 | 88 | 30 | 1 | C→U | CGG→UGG | R→W | 8830 |
| rps4 | 175 | 59 | 1 | C→U | CCG→UCG | P→S | 17559 |
| rps4 | 226 | 76 | 1 | C→U | CCC→UCC | P→S | 22676 |
| rps4 | 235 | 79 | 1 | C→U | CAU→UAU | H→Y | 23579 |
| rps4 | 299 | 100 | 2 | C→U | CCA→CUA | P→L | 299100 |
| rps4 | 308 | 103 | 2 | C→U | CCA→CUA | P→L | 308103 |
| rps4 | 332 | 111 | 2 | C→U | CCG→CUG | P→L | 332111 |
| rps4 | 377 | 126 | 2 | C→U | CCG→CUG | P→L | 377126 |
| rps4 | 524 | 175 | 2 | C→U | UCA→UUA | S→L | 524175 |
| rps4 | 956 | 319 | 2 | C→U | UCG→UUG | S→L | 956319 |
| rps4 | 967 | 323 | 1 | C→U | CAU→UAU | H→Y | 967323 |
| rps4 | 992 | 331 | 2 | C→U | UCU→UUU | S→F | 992331 |
| rps4 | 1043 | 348 | 2 | C→U | CCA→CUA | P→L | 1043348 |
| rps4 | 1052 | 351 | 2 | C→U | CCU→CUU | P→L | 1052351 |
| rps4 | 1057 | 353 | 1 | C→U | CGG→UGG | R→W | 1057353 |
