# Supplementary material for: Mitochondrial genome of Isatis indigotica reveals repeat-mediated recombination and phylogenetic insights in Cruciferae
Source: Front Plant Sci. 2025 Oct 15;16:1655810. doi: 10.3389/fpls.2025.1655810 (PMC12568568; doi:10.3389/fpls.2025.1655810)
Supplement: Supplementary file 10 [file Table10.docx]

**Table S8 | Classification and Frequency of RNA Editing Events by Amino Acid Property Transitions in the Mitogenome.**

| **Type** | **RNA-editing** | **Number** | **Percentage** |
| --- | --- | --- | --- |
| hydrophilic- hydrophilic | CAT(H) → TAT (Y) | 14 |  |
|  | CAC (H) → TAC (Y) | 6 |  |
|  | CGC (R) → TGC (C) | 7 |  |
|  | CGT (R) → TGT (C) | 23 |  |
|  | total | 50 | 14.00% |
| hydrophilic- hydrophobic | ACA (T) → ATA (I) | 2 |  |
|  | ACC (T) → ATC (I) | 1 |  |
|  | ACT (T) → ATT (I) | 5 |  |
|  | ACG (T) → ATG (M) | 2 |  |
|  | CGG (R) → TGG (W) | 27 |  |
|  | TCA (S) → TTA (L) | 50 |  |
|  | TCG (S) → TTG (L) | 39 |  |
|  | TCC (S) → TTC (F) | 13 |  |
|  | TCT (S) → TTT (F) | 35 |  |
|  | total | 174 | 48.74% |
| hydrophobic- hydrophilic | CCA (P) → TCA (S) | 5 |  |
|  | CCC (P) → TCC (S) | 6 |  |
|  | CCG (P) → TCG (S) | 4 |  |
|  | CCT (P) → TCT (S) | 15 |  |
|  | total | 30 | 8.40% |
| hydrophobic- hydrophobic | CCA (P) → CTA (L) | 31 |  |
|  | CCC (P) → CTC (L) | 7 |  |
|  | CCG (P) → CTG (L) | 18 |  |
|  | CCT (P) → CTT (L) | 22 |  |
|  | CCC (P) → TTC (F) | 0 |  |
|  | CCT (P) → TTT (F) | 0 |  |
|  | CTC (L) → TTC (F) | 5 |  |
|  | CTT (L) → TTT (F) | 8 |  |
|  | GCA (A) → GTA (V) | 2 |  |
|  | GCC (A) → GTC (V) | 4 |  |
|  | GCG (A) → GTG (V) | 2 |  |
|  | GCT (A) → GTT (V) | 2 |  |
|  | total | 101 | 28.29% |
| hydrophilic-stop | CAA (Q) → TAA (*) | 1 |  |
|  | CGA (R) → TGA (*) | 1 |  |
|  | total | 2 | 0.56% |
|  | all | 357 | 100.00% |
