# Supplementary material for: Mitochondrial genome of Isatis indigotica reveals repeat-mediated recombination and phylogenetic insights in Cruciferae
Source: Front Plant Sci. 2025 Oct 15;16:1655810. doi: 10.3389/fpls.2025.1655810 (PMC12568568; doi:10.3389/fpls.2025.1655810)
Supplement: Supplementary file 11 [file Table11.docx]

**Table S9 | Comparative Analysis of Mitogenome Similarity with Related Species Based on Sequence Alignment Metrics.**

| **query** | **database** | **identity (%)** | **alignment** | **mismatch** | **gap** | **q. start** | **q. end** | **d. start** | **d. end** | **e-value** | **score** |
| --- | --- | --- | --- | --- | --- | --- | --- | --- | --- | --- | --- |
| mtDNA | NC_058313.1 | 94.451 | 3658 | 153 | 24 | 71998 | 75625 | 315790 | 312153 | 0 | 5585 |
| mtDNA | NC_058313.1 | 91.145 | 2891 | 144 | 43 | 155664 | 158470 | 163810 | 160948 | 0 | 3818 |
| mtDNA | NC_058313.1 | 92.174 | 2696 | 140 | 31 | 202171 | 204832 | 56970 | 59628 | 0 | 3744 |
| mtDNA | NC_058313.1 | 92.139 | 2697 | 140 | 31 | 202171 | 204832 | 350505 | 347846 | 0 | 3740 |
| mtDNA | NC_058313.1 | 92.222 | 2507 | 141 | 18 | 69162 | 71639 | 318583 | 316102 | 0 | 3500 |
| mtDNA | NC_058313.1 | 89.735 | 2601 | 191 | 28 | 122554 | 125094 | 5626 | 3042 | 0 | 3254 |
| mtDNA | NC_058313.1 | 89.735 | 2601 | 191 | 28 | 122554 | 125094 | 104384 | 101800 | 0 | 3254 |
| mtDNA | NC_058313.1 | 89.355 | 2386 | 150 | 37 | 20507 | 22855 | 23396 | 21078 | 0 | 2904 |
| mtDNA | NC_058313.1 | 89.355 | 2386 | 150 | 35 | 20507 | 22855 | 224163 | 226481 | 0 | 2904 |
| mtDNA | NC_058313.1 | 91.059 | 2002 | 115 | 37 | 17020 | 18993 | 26973 | 25008 | 0 | 2647 |
| mtDNA | NC_058313.1 | 91.059 | 2002 | 115 | 36 | 17020 | 18993 | 220586 | 222551 | 0 | 2647 |
| mtDNA | NC_058313.1 | 95.253 | 1622 | 73 | 4 | 245302 | 246920 | 66066 | 67686 | 0 | 2566 |
| mtDNA | NC_058313.1 | 94.098 | 1508 | 83 | 1 | 171349 | 172850 | 17046 | 18553 | 0 | 2287 |
| mtDNA | NC_058313.1 | 94.098 | 1508 | 83 | 1 | 171349 | 172850 | 230513 | 229006 | 0 | 2287 |
| mtDNA | NC_058313.1 | 93.631 | 1523 | 77 | 6 | 176016 | 177538 | 178085 | 176583 | 0 | 2257 |
| mtDNA | NC_058313.1 | 97.641 | 1314 | 31 | 0 | 105888 | 107201 | 324339 | 325652 | 0 | 2255 |
| mtDNA | NC_058313.1 | 94.583 | 1403 | 56 | 11 | 232259 | 233652 | 273921 | 272530 | 0 | 2152 |
| mtDNA | NC_058313.1 | 89.557 | 1714 | 84 | 39 | 159013 | 160687 | 160292 | 158635 | 0 | 2085 |
| mtDNA | NC_058313.1 | 91.733 | 1512 | 75 | 21 | 15302 | 16806 | 28448 | 26980 | 0 | 2054 |
| mtDNA | NC_058313.1 | 91.733 | 1512 | 75 | 21 | 15302 | 16806 | 219111 | 220579 | 0 | 2054 |
| mtDNA | NC_058313.1 | 94.44 | 1349 | 36 | 9 | 107335 | 108656 | 325654 | 326990 | 0 | 2039 |
| mtDNA | NC_058313.1 | 93.106 | 1349 | 68 | 13 | 200728 | 202067 | 55594 | 56926 | 0 | 1953 |
| mtDNA | NC_058313.1 | 93.165 | 1346 | 67 | 13 | 200731 | 202067 | 351878 | 350549 | 0 | 1953 |
| mtDNA | NC_058313.1 | 92.993 | 1313 | 87 | 5 | 33439 | 34750 | 299537 | 300845 | 0 | 1910 |
| mtDNA | NC_058313.1 | 92.993 | 1313 | 87 | 5 | 33439 | 34750 | 334009 | 335317 | 0 | 1910 |
| mtDNA | NC_058313.1 | 94.512 | 1239 | 56 | 6 | 188411 | 189641 | 266204 | 267438 | 0 | 1901 |
| mtDNA | NC_058313.1 | 90.582 | 1306 | 93 | 13 | 127579 | 128854 | 279907 | 278602 | 0 | 1703 |
| mtDNA | NC_058313.1 | 95.661 | 1037 | 40 | 3 | 41785 | 42821 | 115671 | 116702 | 0 | 1661 |
| mtDNA | NC_058313.1 | 93.49 | 1106 | 50 | 12 | 179104 | 180192 | 239473 | 240573 | 0 | 1624 |
| mtDNA | NC_058313.1 | 93.792 | 1047 | 52 | 7 | 233705 | 234747 | 272427 | 271390 | 0 | 1561 |
| mtDNA | NC_058313.1 | 91.545 | 1100 | 65 | 17 | 199299 | 200383 | 53933 | 55019 | 0 | 1491 |
| mtDNA | NC_058313.1 | 91.613 | 1097 | 64 | 17 | 199302 | 200383 | 353539 | 352456 | 0 | 1491 |
| mtDNA | NC_058313.1 | 90.668 | 1093 | 81 | 10 | 235437 | 236520 | 320019 | 321099 | 0 | 1434 |
| mtDNA | NC_058313.1 | 89.783 | 1106 | 59 | 25 | 185018 | 186111 | 47059 | 48122 | 0 | 1367 |
| mtDNA | NC_058313.1 | 89.783 | 1106 | 59 | 22 | 185018 | 186111 | 360416 | 359353 | 0 | 1367 |
| mtDNA | NC_058313.1 | 94.072 | 911 | 35 | 10 | 47474 | 48366 | 332101 | 331192 | 0 | 1365 |
| mtDNA | NC_058313.1 | 96.581 | 819 | 19 | 3 | 48409 | 49227 | 330947 | 330138 | 0 | 1349 |
| mtDNA | NC_058313.1 | 93.174 | 923 | 39 | 12 | 248873 | 249792 | 120087 | 120988 | 0 | 1334 |
| mtDNA | NC_058313.1 | 95.053 | 849 | 36 | 3 | 11313 | 12159 | 263871 | 264715 | 0 | 1330 |
| mtDNA | NC_058313.1 | 91.954 | 957 | 55 | 9 | 68187 | 69136 | 319516 | 318575 | 0 | 1321 |
| mtDNA | NC_058313.1 | 93.318 | 898 | 45 | 5 | 228371 | 229256 | 11271 | 10377 | 0 | 1312 |
| mtDNA | NC_058313.1 | 93.318 | 898 | 45 | 5 | 228371 | 229256 | 110029 | 109135 | 0 | 1312 |
| mtDNA | NC_058313.1 | 94.938 | 810 | 37 | 4 | 55702 | 56508 | 266187 | 265379 | 0 | 1266 |
| mtDNA | NC_058313.1 | 93.594 | 843 | 48 | 3 | 207004 | 207842 | 165135 | 164295 | 0 | 1253 |
| mtDNA | NC_058313.1 | 93.594 | 843 | 48 | 3 | 119289 | 120127 | 165135 | 164295 | 0 | 1253 |
| mtDNA | NC_058313.1 | 94.717 | 795 | 40 | 2 | 175046 | 175839 | 178887 | 178094 | 0 | 1234 |
| mtDNA | NC_058313.1 | 89.409 | 982 | 90 | 8 | 147284 | 148257 | 41397 | 40422 | 0 | 1225 |
| mtDNA | NC_058313.1 | 89.479 | 979 | 89 | 8 | 147284 | 148254 | 366078 | 367050 | 0 | 1225 |
| mtDNA | NC_058313.1 | 88.621 | 1037 | 75 | 16 | 210269 | 211275 | 5626 | 4603 | 0 | 1221 |
| mtDNA | NC_058313.1 | 88.621 | 1037 | 75 | 16 | 210269 | 211275 | 104384 | 103361 | 0 | 1221 |
| mtDNA | NC_058313.1 | 91.314 | 898 | 63 | 6 | 125192 | 126081 | 10342 | 9452 | 0 | 1212 |
| mtDNA | NC_058313.1 | 91.314 | 898 | 63 | 6 | 125192 | 126081 | 109100 | 108210 | 0 | 1212 |
| mtDNA | NC_058313.1 | 96.28 | 699 | 26 | 0 | 248082 | 248780 | 118844 | 119542 | 0 | 1147 |
| mtDNA | NC_058313.1 | 93.582 | 779 | 33 | 6 | 190370 | 191147 | 181724 | 180962 | 0 | 1146 |
| mtDNA | NC_058313.1 | 94.071 | 759 | 30 | 5 | 50632 | 51389 | 125644 | 126388 | 0 | 1138 |
| mtDNA | NC_058313.1 | 93.003 | 786 | 41 | 4 | 258836 | 259621 | 1838 | 2609 | 0 | 1134 |
| mtDNA | NC_058313.1 | 93.003 | 786 | 41 | 4 | 258836 | 259621 | 100596 | 101367 | 0 | 1134 |
| mtDNA | NC_058313.1 | 91.787 | 828 | 41 | 14 | 32573 | 33378 | 298562 | 299384 | 0 | 1127 |
| mtDNA | NC_058313.1 | 91.787 | 828 | 41 | 14 | 32573 | 33378 | 333034 | 333856 | 0 | 1127 |
| mtDNA | NC_058313.1 | 92.387 | 775 | 47 | 10 | 256780 | 257548 | 340842 | 341610 | 0 | 1094 |
| mtDNA | NC_058313.1 | 89.929 | 844 | 42 | 14 | 183122 | 183950 | 44580 | 45395 | 0 | 1048 |
| mtDNA | NC_058313.1 | 89.929 | 844 | 42 | 14 | 183122 | 183950 | 362895 | 362080 | 0 | 1048 |
| mtDNA | NC_058313.1 | 90.85 | 765 | 70 | 0 | 60110 | 60874 | 167517 | 166753 | 0 | 1026 |
| mtDNA | NC_058313.1 | 96.817 | 597 | 19 | 0 | 190370 | 190966 | 69123 | 68527 | 0 | 998 |
| mtDNA | NC_058313.1 | 87.13 | 878 | 71 | 22 | 209420 | 210271 | 6978 | 6117 | 0 | 957 |
| mtDNA | NC_058313.1 | 87.13 | 878 | 71 | 22 | 121705 | 122556 | 6978 | 6117 | 0 | 957 |
| mtDNA | NC_058313.1 | 87.13 | 878 | 71 | 22 | 209420 | 210271 | 105736 | 104875 | 0 | 957 |
| mtDNA | NC_058313.1 | 87.13 | 878 | 71 | 22 | 121705 | 122556 | 105736 | 104875 | 0 | 957 |
| mtDNA | NC_058313.1 | 95.017 | 582 | 29 | 0 | 88110 | 88691 | 65848 | 65267 | 0 | 915 |
| mtDNA | NC_058313.1 | 85.177 | 904 | 83 | 27 | 19535 | 20412 | 24287 | 23409 | 0 | 880 |
| mtDNA | NC_058313.1 | 85.177 | 904 | 83 | 27 | 19535 | 20412 | 223272 | 224150 | 0 | 880 |
| mtDNA | NC_058313.1 | 93.287 | 581 | 29 | 7 | 184282 | 184856 | 46203 | 46779 | 0 | 848 |
| mtDNA | NC_058313.1 | 93.849 | 569 | 25 | 7 | 184294 | 184856 | 361260 | 360696 | 0 | 848 |
| mtDNA | NC_058313.1 | 89.954 | 647 | 56 | 6 | 235877 | 236520 | 95693 | 96333 | 0 | 826 |
| mtDNA | NC_058313.1 | 89.954 | 647 | 56 | 6 | 235877 | 236520 | 208294 | 208934 | 0 | 826 |
| mtDNA | NC_058313.1 | 91.137 | 519 | 25 | 10 | 22906 | 23408 | 20233 | 19720 | 0 | 684 |
| mtDNA | NC_058313.1 | 91.137 | 519 | 25 | 10 | 22906 | 23408 | 227326 | 227839 | 0 | 684 |
| mtDNA | NC_058313.1 | 84.502 | 542 | 27 | 13 | 19035 | 19535 | 24996 | 24471 | 3.63E-135 | 483 |
| mtDNA | NC_058313.1 | 84.502 | 542 | 27 | 13 | 19035 | 19535 | 222563 | 223088 | 3.63E-135 | 483 |
| mtDNA | NC_058313.1 | 81.818 | 572 | 62 | 20 | 196556 | 197096 | 286103 | 286663 | 6.16E-123 | 442 |
| mtDNA | NC_067879.1 | 92.652 | 4110 | 205 | 45 | 200728 | 204797 | 110185 | 114237 | 0 | 5827 |
| mtDNA | NC_067879.1 | 94.737 | 3249 | 131 | 17 | 72483 | 75712 | 74102 | 70875 | 0 | 5016 |
| mtDNA | NC_067879.1 | 92.662 | 2521 | 152 | 17 | 175044 | 177557 | 174332 | 171838 | 0 | 3600 |
| mtDNA | NC_067879.1 | 90.272 | 2827 | 138 | 50 | 155634 | 158361 | 37122 | 34334 | 0 | 3570 |
| mtDNA | NC_067879.1 | 92.723 | 2501 | 137 | 21 | 69162 | 71639 | 166773 | 169251 | 0 | 3568 |
| mtDNA | NC_067879.1 | 89.271 | 2591 | 217 | 24 | 122552 | 125113 | 181191 | 178633 | 0 | 3188 |
| mtDNA | NC_067879.1 | 95.136 | 1686 | 79 | 3 | 245305 | 246989 | 63722 | 65405 | 0 | 2656 |
| mtDNA | NC_067879.1 | 97.595 | 1414 | 33 | 1 | 105888 | 107300 | 145575 | 146988 | 0 | 2422 |
| mtDNA | NC_067879.1 | 97.727 | 1408 | 31 | 1 | 105894 | 107300 | 274327 | 272920 | 0 | 2422 |
| mtDNA | NC_067879.1 | 93.91 | 1527 | 87 | 1 | 171330 | 172850 | 85096 | 86622 | 0 | 2300 |
| mtDNA | NC_067879.1 | 90.476 | 1722 | 92 | 32 | 231972 | 233652 | 6252 | 4562 | 0 | 2206 |
| mtDNA | NC_067879.1 | 93.664 | 1452 | 65 | 13 | 15039 | 16465 | 243461 | 244910 | 0 | 2146 |
| mtDNA | NC_067879.1 | 95.288 | 1252 | 47 | 3 | 127573 | 128818 | 177447 | 176202 | 0 | 1975 |
| mtDNA | NC_067879.1 | 90.195 | 1540 | 91 | 22 | 119262 | 120768 | 300561 | 302073 | 0 | 1953 |
| mtDNA | NC_067879.1 | 90.195 | 1540 | 91 | 22 | 206977 | 208483 | 300561 | 302073 | 0 | 1953 |
| mtDNA | NC_067879.1 | 93.213 | 1326 | 86 | 4 | 33439 | 34762 | 137199 | 135876 | 0 | 1947 |
| mtDNA | NC_067879.1 | 93.45 | 1313 | 84 | 2 | 33439 | 34751 | 282709 | 284019 | 0 | 1947 |
| mtDNA | NC_067879.1 | 91.512 | 1402 | 85 | 17 | 16488 | 17875 | 244904 | 246285 | 0 | 1899 |
| mtDNA | NC_067879.1 | 92.526 | 1338 | 69 | 11 | 179106 | 180428 | 233706 | 232385 | 0 | 1888 |
| mtDNA | NC_067879.1 | 90.378 | 1455 | 65 | 31 | 159013 | 160447 | 33687 | 32288 | 0 | 1842 |
| mtDNA | NC_067879.1 | 92.514 | 1269 | 67 | 13 | 41785 | 43039 | 19054 | 17800 | 0 | 1792 |
| mtDNA | NC_067879.1 | 95.664 | 1084 | 35 | 7 | 107296 | 108378 | 147191 | 148263 | 0 | 1731 |
| mtDNA | NC_067879.1 | 95.745 | 1081 | 34 | 7 | 107299 | 108378 | 272714 | 271645 | 0 | 1731 |
| mtDNA | NC_067879.1 | 92.066 | 1210 | 79 | 6 | 188411 | 189620 | 115634 | 114442 | 0 | 1687 |
| mtDNA | NC_067879.1 | 96.341 | 984 | 28 | 4 | 27240 | 28217 | 99068 | 98087 | 0 | 1611 |
| mtDNA | NC_067879.1 | 93.352 | 1098 | 45 | 15 | 199299 | 200378 | 108396 | 109483 | 0 | 1598 |
| mtDNA | NC_067879.1 | 91.791 | 1072 | 59 | 11 | 233670 | 234731 | 4314 | 3262 | 0 | 1465 |
| mtDNA | NC_067879.1 | 95.125 | 923 | 38 | 3 | 248873 | 249790 | 189454 | 190374 | 0 | 1448 |
| mtDNA | NC_067879.1 | 88.824 | 1199 | 93 | 20 | 256376 | 257559 | 55789 | 56961 | 0 | 1434 |
| mtDNA | NC_067879.1 | 94.354 | 921 | 41 | 7 | 47472 | 48384 | 258470 | 257553 | 0 | 1402 |
| mtDNA | NC_067879.1 | 91.772 | 948 | 57 | 13 | 68194 | 69136 | 165850 | 166781 | 0 | 1299 |
| mtDNA | NC_067879.1 | 90.581 | 998 | 64 | 15 | 125187 | 126169 | 224470 | 225452 | 0 | 1295 |
| mtDNA | NC_067879.1 | 93.258 | 890 | 39 | 12 | 11286 | 12159 | 250816 | 251700 | 0 | 1291 |
| mtDNA | NC_067879.1 | 89.882 | 1018 | 81 | 11 | 210267 | 211275 | 181191 | 180187 | 0 | 1290 |
| mtDNA | NC_067879.1 | 93.961 | 828 | 34 | 4 | 50630 | 51451 | 304381 | 303564 | 0 | 1238 |
| mtDNA | NC_067879.1 | 91.657 | 899 | 46 | 14 | 258735 | 259621 | 139140 | 138259 | 0 | 1218 |
| mtDNA | NC_067879.1 | 91.741 | 896 | 45 | 14 | 258735 | 259618 | 280768 | 281646 | 0 | 1218 |
| mtDNA | NC_067879.1 | 96.97 | 726 | 16 | 1 | 48471 | 49196 | 257529 | 256810 | 0 | 1214 |
| mtDNA | NC_067879.1 | 90.979 | 909 | 69 | 2 | 228491 | 229390 | 290551 | 291455 | 0 | 1212 |
| mtDNA | NC_067879.1 | 93.423 | 821 | 41 | 7 | 55702 | 56519 | 115651 | 116461 | 0 | 1205 |
| mtDNA | NC_067879.1 | 88.269 | 1057 | 59 | 16 | 183122 | 184168 | 220950 | 221951 | 0 | 1205 |
| mtDNA | NC_067879.1 | 93.643 | 818 | 29 | 9 | 190332 | 191147 | 15704 | 16500 | 0 | 1201 |
| mtDNA | NC_067879.1 | 94.452 | 775 | 43 | 0 | 60098 | 60872 | 116901 | 117675 | 0 | 1194 |
| mtDNA | NC_067879.1 | 89.852 | 877 | 69 | 15 | 147388 | 148247 | 164255 | 165128 | 0 | 1109 |
| mtDNA | NC_067879.1 | 88.359 | 859 | 74 | 16 | 209420 | 210271 | 182506 | 181667 | 0 | 1009 |
| mtDNA | NC_067879.1 | 88.359 | 859 | 74 | 16 | 121705 | 122556 | 182506 | 181667 | 0 | 1009 |
| mtDNA | NC_067879.1 | 88.181 | 863 | 51 | 21 | 184992 | 185836 | 222667 | 223496 | 0 | 981 |
| mtDNA | NC_067879.1 | 94.728 | 588 | 25 | 3 | 248192 | 248779 | 188317 | 188898 | 0 | 909 |
| mtDNA | NC_067879.1 | 95.246 | 568 | 23 | 1 | 32811 | 33378 | 137926 | 137363 | 0 | 896 |
| mtDNA | NC_067879.1 | 95.246 | 568 | 23 | 1 | 32811 | 33378 | 281982 | 282545 | 0 | 896 |
| mtDNA | NC_067879.1 | 87.516 | 777 | 80 | 11 | 235725 | 236493 | 44700 | 45467 | 0 | 881 |
| mtDNA | NC_067879.1 | 87.948 | 614 | 50 | 13 | 41181 | 41784 | 20984 | 20385 | 0 | 702 |
| mtDNA | NC_067879.1 | 84.848 | 693 | 55 | 27 | 160674 | 161352 | 32130 | 31474 | 0 | 652 |
| mtDNA | NC_067879.1 | 79.825 | 570 | 61 | 26 | 57665 | 58222 | 204277 | 204804 | 3.13E-100 | 366 |
| mtDNA | PP916044.1 | 99.826 | 69059 | 66 | 10 | 54513 | 123560 | 144738 | 75723 | 0 | ##### |
| mtDNA | PP916044.1 | 99.917 | 44672 | 10 | 1 | 117232 | 161903 | 207278 | 251922 | 0 | 82263 |
| mtDNA | PP916044.1 | 99.872 | 32016 | 5 | 4 | 204947 | 236956 | 82024 | 50039 | 0 | 58863 |
| mtDNA | PP916044.1 | 99.754 | 25247 | 27 | 4 | 186030 | 211275 | 188367 | 213579 | 0 | 46246 |
| mtDNA | PP916044.1 | 99.668 | 24376 | 62 | 7 | 30755 | 55113 | 26266 | 50639 | 0 | 44547 |
| mtDNA | PP916044.1 | 99.953 | 21300 | 5 | 1 | 4322 | 25616 | 4978 | 26277 | 0 | 39273 |
| mtDNA | PP916044.1 | 99.958 | 16803 | 6 | 1 | 168967 | 185768 | 171564 | 188366 | 0 | 30989 |
| mtDNA | PP916044.1 | 100 | 15612 | 0 | 0 | 245253 | 260864 | 153003 | 168614 | 0 | 28831 |
| mtDNA | PP916044.1 | 99.899 | 8873 | 1 | 2 | 236358 | 245226 | 144140 | 153008 | 0 | 16329 |
| mtDNA | PP916044.1 | 99.653 | 4324 | 10 | 2 | 1 | 4319 | 739 | 5062 | 0 | 7897 |
| mtDNA | PP916044.1 | 100 | 3690 | 0 | 0 | 161904 | 165593 | 167877 | 171566 | 0 | 6815 |
| mtDNA | PP916044.1 | 99.864 | 738 | 1 | 0 | 161904 | 162641 | 1 | 738 | 0 | 1358 |
| mtDNA | PP916044.1 | 99.864 | 738 | 1 | 0 | 260127 | 260864 | 1 | 738 | 0 | 1358 |
| mtDNA | PP916044.1 | 100 | 583 | 0 | 0 | 102323 | 102905 | 193610 | 193028 | 0 | 1077 |
| mtDNA | PP916044.1 | 99.657 | 583 | 2 | 0 | 190691 | 191273 | 96351 | 96933 | 0 | 1066 |
| mtDNA | NC_021092.1 | 94.651 | 3141 | 96 | 28 | 72540 | 75628 | 121111 | 117991 | 0 | 4804 |
| mtDNA | NC_021092.1 | 93.331 | 2699 | 117 | 25 | 200730 | 203393 | 358481 | 361151 | 0 | 3928 |
| mtDNA | NC_021092.1 | 92.753 | 2470 | 124 | 25 | 69193 | 71638 | 67509 | 65071 | 0 | 3518 |
| mtDNA | NC_021092.1 | 89.988 | 2547 | 141 | 52 | 16488 | 18993 | 87553 | 90026 | 0 | 3186 |
| mtDNA | NC_021092.1 | 89.777 | 2553 | 124 | 55 | 155664 | 158151 | 100897 | 98417 | 0 | 3142 |
| mtDNA | NC_021092.1 | 90.747 | 2356 | 164 | 17 | 122815 | 125125 | 227309 | 229655 | 0 | 3094 |
| mtDNA | NC_021092.1 | 87.423 | 2584 | 182 | 61 | 19535 | 22057 | 90766 | 93267 | 0 | 2839 |
| mtDNA | NC_021092.1 | 95.473 | 1679 | 74 | 2 | 245305 | 246982 | 219782 | 221459 | 0 | 2678 |
| mtDNA | NC_021092.1 | 94 | 1550 | 64 | 9 | 176023 | 177557 | 366414 | 364879 | 0 | 2320 |
| mtDNA | NC_021092.1 | 93.75 | 1520 | 93 | 2 | 171330 | 172847 | 175251 | 176770 | 0 | 2279 |
| mtDNA | NC_021092.1 | 94.408 | 1359 | 53 | 7 | 127522 | 128868 | 265012 | 266359 | 0 | 2067 |
| mtDNA | NC_021092.1 | 91.732 | 1524 | 68 | 20 | 188411 | 189908 | 195504 | 196995 | 0 | 2063 |
| mtDNA | NC_021092.1 | 93.782 | 1367 | 41 | 14 | 107324 | 108657 | 256814 | 255459 | 0 | 2013 |
| mtDNA | NC_021092.1 | 92.34 | 1436 | 63 | 22 | 125215 | 126613 | 263311 | 264736 | 0 | 1999 |
| mtDNA | NC_021092.1 | 92.202 | 1385 | 99 | 7 | 105888 | 107270 | 258211 | 256834 | 0 | 1951 |
| mtDNA | NC_021092.1 | 93.204 | 1339 | 65 | 15 | 179106 | 180425 | 376285 | 377616 | 0 | 1945 |
| mtDNA | NC_021092.1 | 91.233 | 1460 | 73 | 24 | 232219 | 233652 | 150169 | 148739 | 0 | 1936 |
| mtDNA | NC_021092.1 | 90.706 | 1474 | 84 | 25 | 203383 | 204831 | 361358 | 362803 | 0 | 1914 |
| mtDNA | NC_021092.1 | 94.619 | 1208 | 54 | 5 | 15259 | 16465 | 86362 | 87559 | 0 | 1860 |
| mtDNA | NC_021092.1 | 91.892 | 1295 | 97 | 8 | 33438 | 34726 | 126715 | 128007 | 0 | 1803 |
| mtDNA | NC_021092.1 | 91.61 | 1168 | 63 | 10 | 185019 | 186167 | 224871 | 226022 | 0 | 1581 |
| mtDNA | NC_021092.1 | 91.863 | 1106 | 47 | 21 | 199299 | 200383 | 356830 | 357913 | 0 | 1504 |
| mtDNA | NC_021092.1 | 94.809 | 944 | 26 | 9 | 159013 | 159942 | 97173 | 96239 | 0 | 1450 |
| mtDNA | NC_021092.1 | 91.386 | 1068 | 74 | 6 | 235422 | 236480 | 314541 | 315599 | 0 | 1447 |
| mtDNA | NC_021092.1 | 93.52 | 895 | 42 | 7 | 228368 | 229252 | 396147 | 397035 | 0 | 1317 |
| mtDNA | NC_021092.1 | 96.97 | 759 | 17 | 6 | 48471 | 49227 | 184554 | 183800 | 0 | 1269 |
| mtDNA | NC_021092.1 | 93.262 | 846 | 51 | 4 | 11281 | 12124 | 241107 | 241948 | 0 | 1242 |
| mtDNA | NC_021092.1 | 90.393 | 968 | 49 | 20 | 68187 | 69136 | 68463 | 67522 | 0 | 1232 |
| mtDNA | NC_021092.1 | 90.572 | 944 | 43 | 20 | 248873 | 249782 | 14291 | 15222 | 0 | 1208 |
| mtDNA | NC_021092.1 | 90.105 | 950 | 45 | 26 | 47442 | 48365 | 185526 | 184600 | 0 | 1188 |
| mtDNA | NC_021092.1 | 93.095 | 811 | 52 | 4 | 55701 | 56508 | 195488 | 194679 | 0 | 1184 |
| mtDNA | NC_021092.1 | 97.08 | 685 | 11 | 4 | 50630 | 51309 | 291084 | 291764 | 0 | 1146 |
| mtDNA | NC_021092.1 | 93.642 | 755 | 30 | 7 | 233674 | 234416 | 148482 | 147734 | 0 | 1112 |
| mtDNA | NC_021092.1 | 94.825 | 715 | 33 | 1 | 175115 | 175829 | 370449 | 369739 | 0 | 1112 |
| mtDNA | NC_021092.1 | 90.151 | 863 | 70 | 7 | 147403 | 148260 | 171482 | 170630 | 0 | 1109 |
| mtDNA | NC_021092.1 | 91.729 | 798 | 35 | 14 | 183178 | 183967 | 222619 | 223393 | 0 | 1079 |
| mtDNA | NC_021092.1 | 88.103 | 933 | 72 | 18 | 121651 | 122557 | 226017 | 226936 | 0 | 1072 |
| mtDNA | NC_021092.1 | 88.103 | 933 | 72 | 18 | 209366 | 210272 | 226017 | 226936 | 0 | 1072 |
| mtDNA | NC_021092.1 | 95.06 | 668 | 28 | 1 | 190328 | 190990 | 17659 | 16992 | 0 | 1046 |
| mtDNA | NC_021092.1 | 90.535 | 803 | 56 | 9 | 256767 | 257557 | 215344 | 214550 | 0 | 1044 |
| mtDNA | NC_021092.1 | 97.078 | 616 | 18 | 0 | 248164 | 248779 | 13196 | 13811 | 0 | 1038 |
| mtDNA | NC_021092.1 | 91.645 | 754 | 47 | 9 | 258871 | 259621 | 181882 | 181142 | 0 | 1029 |
| mtDNA | NC_021092.1 | 91.459 | 761 | 47 | 6 | 210530 | 211275 | 227309 | 228066 | 0 | 1029 |
| mtDNA | NC_021092.1 | 95.49 | 643 | 29 | 0 | 207126 | 207768 | 12604 | 11962 | 0 | 1027 |
| mtDNA | NC_021092.1 | 95.49 | 643 | 29 | 0 | 119411 | 120053 | 12604 | 11962 | 0 | 1027 |
| mtDNA | NC_021092.1 | 90.621 | 757 | 62 | 1 | 60112 | 60868 | 288347 | 289094 | 0 | 996 |
| mtDNA | NC_021092.1 | 91.737 | 714 | 22 | 9 | 223081 | 223784 | 165179 | 164493 | 0 | 957 |
| mtDNA | NC_021092.1 | 91.111 | 720 | 31 | 17 | 22850 | 23550 | 94684 | 95389 | 0 | 944 |
| mtDNA | NC_021092.1 | 87.471 | 854 | 48 | 28 | 57933 | 58757 | 211021 | 211844 | 0 | 929 |
| mtDNA | NC_021092.1 | 94.774 | 574 | 21 | 2 | 32805 | 33378 | 125938 | 126502 | 0 | 885 |
| mtDNA | NC_021092.1 | 92.27 | 608 | 33 | 8 | 22252 | 22855 | 93245 | 93842 | 0 | 850 |
| mtDNA | NC_021092.1 | 93.092 | 579 | 23 | 9 | 184291 | 184861 | 224194 | 224763 | 0 | 832 |
| mtDNA | NC_021092.1 | 83.81 | 840 | 69 | 34 | 158176 | 158961 | 98365 | 97539 | 0 | 736 |
| mtDNA | NC_021092.1 | 81.569 | 548 | 37 | 30 | 19035 | 19533 | 90038 | 90570 | 1.91E-108 | 394 |
